# Complete Genome Sequence of *Borrelia afzelii* K78 and Comparative Genome Analysis

**DOI:** 10.1371/journal.pone.0120548

**Published:** 2015-03-23

**Authors:** Wolfgang Schüler, Ignas Bunikis, Jacqueline Weber-Lehman, Pär Comstedt, Sabrina Kutschan-Bunikis, Gerold Stanek, Jutta Huber, Andreas Meinke, Sven Bergström, Urban Lundberg

**Affiliations:** 1 Valneva Austria GmbH, Vienna, Austria; 2 Department of Molecular Biology, Umeå University, Umeå, Sweden; 3 Eurofins Genomics GmbH, Ebersberg, Germany; 4 Medical University of Vienna, Institute for Hygiene and Applied Immunology, Vienna, Austria; University of Kentucky College of Medicine, UNITED STATES

## Abstract

The main *Borrelia* species causing Lyme borreliosis in Europe and Asia are *Borrelia afzelii*, *B*. *garinii*, *B*. *burgdorferi* and *B*. *bavariensis*. This is in contrast to the United States, where infections are exclusively caused by *B*. *burgdorferi*. Until to date the genome sequences of four *B*. *afzelii* strains, of which only two include the numerous plasmids, are available. In order to further assess the genetic diversity of *B*. *afzelii*, the most common species in Europe, responsible for the large variety of clinical manifestations of Lyme borreliosis, we have determined the full genome sequence of the *B*. *afzelii* strain K78, a clinical isolate from Austria. The K78 genome contains a linear chromosome (905,949 bp) and 13 plasmids (8 linear and 5 circular) together presenting 1,309 open reading frames of which 496 are located on plasmids. With the exception of lp28-8, all linear replicons in their full length including their telomeres have been sequenced. The comparison with the genomes of the four other *B*. *afzelii* strains, ACA-1, PKo, HLJ01 and Tom3107, as well as the one of *B*. *burgdorferi* strain B31, confirmed a high degree of conservation within the linear chromosome of *B*. *afzelii*, whereas plasmid encoded genes showed a much larger diversity. Since some plasmids present in *B*. *burgdorferi* are missing in the *B*. *afzelii* genomes, the corresponding virulence factors of *B*. *burgdorferi* are found in *B*. *afzelii* on other unrelated plasmids. In addition, we have identified a species specific region in the circular plasmid, cp26, which could be used for species determination. Different non-coding RNAs have been located on the *B*. *afzelii* K78 genome, which have not previously been annotated in any of the published *Borrelia* genomes.

## Introduction

Lyme borreliosis (LB) is a major cause of morbidity in temperate climates of the Northern hemisphere. The endemic area covers countries from Portugal in Western Europe to Japan in Eastern Asia and also large parts of the American continent. The highest incidence rates of LB are found in central and Eastern Europe as well as the North Eastern part of the United States. *Borrelia* species causing LB are transmitted by hard ticks (*Ixodes spp*) [1] and the natural reservoirs are typically small mammals like rodents and shrews as well as birds [2]. In the United States LB is caused exclusively by *B*. *burgdorferi* which is in contrast to Europe, where *B*. *afzelii*, *B*. *garinii*, *B*. *burgdorferi* and *B*. *bavariensis* are most predominant. Of these four, *B*. *afzelii* is the most common species found in *Ixodes* ticks [3] and most frequently isolated from LB patients in Europe.

The various LB causing *Borrelia* species are believed to have partially different tissue tropism and therefore distinct pathogenicity and clinical disease patterns. Certain subspecies can also differ in their virulence indicating genetic variability within individual *Borrelia* species. These virulence traits might explain the various disease causing capacities of distinct *Borrelia* species, as well as their ability to colonize and propagate in different tissues. Thus, even though *Borrelia* genomes are relatively similar, the individual species can cause different clinical manifestations of LB: *B*. *burgdorferi* is often associated with arthritis [4], *B*. *garinii* and *B*. *bavariensis* with neuroborreliosis [5] and *B*. *afzelii* with chronic skin conditions [6, 7]. With the increasing availability of genome data from the various *Borrelia* species it might be possible to elucidate the genetic basis for the difference in tropism between the LB causing *Borrelia* species. While the number of genome sequences from *B*. *burgdorferi* strains has grown considerably in the last years [8–10], sequencing of the other species responsible for the majority of LB cases in Europe; *B*. *afzelii*, *B*. *bavariensis* and *B*. *garinii*, is significantly lagging behind.

It has been shown that the *Borrelia* genome is very complex, consisting of a linear chromosome and a large set of both circular and linear plasmids. In addition, it has a low G+C content, e.g. for K78 only 28%, which is at the low end of what is reported for prokaryotic genomes in the GenBank database (14–75%; NCBI, www.ncbi.nlm.nih.gov/genome/browse/). At present, 27 partial or complete genome sequences from different bacterial strains associated with LB are available. The sequences were determined for 14 *B*. *burgdorferi* [8–10], five *B*. *garinii* [11–14], one *B*. *bavariensis* [15], *B*. *valaisiana*, *B*. *spielmanii* [16], and *B*. *finlandensis* sp. nov. [17] each, and four *B*. *afzelii* strains [11, 18–20].

The four *B*. *afzelii* genome sequences so far determined (PKo, ACA-1, HLJ01 and Tom3107) are not all complete [11, 18–20]. For PKo, data from two different sequencing projects of the complete genome have been made available [11, 18]. For Tom3107 the linear chromosome and two plasmids are deposited at GenBank [20] and for HLJ01 only the linear chromosome has yet been published [19], and the sequence data available for the linear chromosome of ACA-1 is only available as two contigs [11]. We report here the complete genome sequence of an Austrian *B*. *afzelii* strain, K78, showing a close relationship to the other *B*. *afzelii* strains. This has allowed us for the first time to compare three European *B*. *afzelii* genome sequences including plasmids and to relate our findings to the chromosomes of a Chinese and a Russian *B*. *afzelii* strain (19, 20] and the *B*. *burgdorferi* strain B31 [8, 9].

## Materials and Methods

### Growth conditions and DNA preparation

*B*. *afzelii* K78 is an isolate from a skin biopsy (primary erythema migrans lesion) from an Austrian Lyme borreliosis (LB) patient [21]. DNA isolated from this strain (passage 5) was used for sequencing. In short, K78 was grown in BSK-II medium [22] supplemented with 6% rabbit serum (Sigma-Aldrich, USA) at 35°C until the cell density reached approximately 10^7^–10^8^ cells/mL. Genomic DNA used to generate the sequence of the linear chromosome was purified using the Wizard Genomic DNA purification Kit (Promega, USA). Plasmid DNA was extracted using QIAGEN Plasmid Midi Kit (Qiagen, Germany) following a protocol adapted for *Borrelia* spp. (http://www.qiagen.com).

### DNA sequencing and genome assembly

The initial genome sequencing was performed by Sanger shotgun sequencing of a pGEM-T Easy library containing 1.5–2.0 kbp inserts of *Borrelia* DNA which was cobalt-hexamine precipitated prior to cloning. The linear chromosome was sequenced to 7.6-fold coverage. Because of the high similarity of the *Borrelia* chromosomes, the sequences were mapped to the *B*. *burgdorferi* B31 chromosome applying the phrap, consed, Staden package and MUMmer software [23–25]. Gaps between the assembled contigs of the chromosome were closed by cloning and primer walking. The initial assembly of plasmids was performed using plasmid sequences obtained from sequencing of the pGEM-T Easy library. To complete the sequences of plasmids, two additional data sets were generated using plasmid DNA as a template. Firstly, a rapid fragment library was sequenced using the 454 pyrosequencing method (Roche, USA) to obtain long reads (Karolinska Institutet, Sweden). Secondly, a 2×50-bp mate-paired library with a mean insert size of 1.5 kbp was additionally sequenced on a SOLiD4 instrument (LifeTechnologies, USA) to generate short reads (Uppsala Genome Center, Sweden). Before *de novo* assembly, data sets were filtered to remove reads containing ambiguities and low quality bases, adapter sequences (454) and reads shorter than 40 (454) or 50 (SOLiD) nucleotides. High predicted plasmid coverage (approximately 2,800-fold) in the SOLiD data set could be achieved and allowed for harsh filtering in order to obtain a data set of very high quality. The 454 reads were assembled using Newbler [26] (Roche). In addition, SOLiD reads were assembled using Velvet [27]. Two assemblies were merged and served as input for HAPS (Hybrid Assembly Pipeline with SOLiD reads) [28]. HAPS uses mate-pair information from SOLiD reads to order and scaffold contigs. Gaps in the obtained draft plasmid sequences were filled either by recursively mapping all reads or by utilizing sequencing data generated earlier by Sanger shotgun sequencing or by primer walking. In addition, reads generated by SOLiD technology were used to find and correct errors in stretches of homopolymeric sequences which are common in *Borrelia* genomes. This was done by mapping SOLiD reads to the draft genome sequence using LifeScope (LifeTechnologies) followed by manual sequence correction. The final assembly was evaluated with the integrative genomics viewer [29]. The use of reads from two different next generation sequencing technologies greatly facilitated scaffolding, gap filling and finishing the *B*. *afzelii* K78 genome sequence.

### Sequence annotation

The open reading frames in the genome were annotated using Glimmer3 (gene prediction) [30], RNAmmer (rRNA identification) [31], and tRNAscan-SE (transfer RNA assignments) [32]. Sequence annotation was matched against UniProt [33], COG [34], CDD [35], TIGRFAM [36], Pfam [37] and Rfam [38]. To optimize the results of the gene prediction with Glimmer3, the translation initiation sites of the annotated genes were analyzed with the TriTISA program [39] and manually compared with the genes of the known annotated *Borrelia* genomes. Further manual refinement included information from InterProScan [40] and alignments against the non-redundant protein sequence database (nr) of NCBI [41] with BLAST.

Sequence visualization and interactive annotation were done with the Artemis software package from Sanger institute [42]. Sequences of the replicons were aligned to the sequences of *B*. *afzelii* ACA-1, PKo, HLJ01 and *B*. *burgdorferi* B31 with the progressive alignment algorithm of Mauve [43] and with the program stretcher of the EMBOSS software package, which applies a Needleman-Wunsch algorithm, modified to allow global alignments of longer sequences [44]. Orthologs were identified with cd-hit sequence clustering [45]. A classification of the predicted proteins into the scheme of paralogous gene families as proposed by Casjens *et al*. [46] was obtained with application of the TribeMCL [47] and spectral clustering algorithms of SCPS (Spectral Clustering of Protein Sequences) [48]. Tandem repeats in the genome were detected with the Tandem Repeat Finder program [49].

The subcellular localization of K78 proteins was predicted with PSORTb [50] (S1 Table). Signal sequences and lipidation signals were identified with SignalP 3.0 [51] and SpLip (spirochaetal lipoprotein prediction tool) [52]. Membrane protein detection was supported with TMHMM transmembrane helix prediction [53].

### Phylogenetic analysis *in silico*

For genomic typing (Table 1) multi locus sequence typing (MLST) allelic profiles were analyzed looking at the housekeeping genes, *clpA*, *clpX*, *nifS*, *pepX*, *pyrG*, *recG*, *rplB* and *uvrA*, according to the procedure of Margos *et al*. [54]. To determine the sequence types, the segments of the concatenated housekeeping genes [55] or of the *ospA* sequences were used as defined in the *B*. *burgdorferi* MLSA database [56].

**Table 1:** Comparison of the sequence types of *B*. *afzelii* strains according to multi locus sequence typing (MLST), and *ospA* and *ospC* typing.

Organism	MLST	*ospA*	*ospC*
Baf_K78	ST335	3 (2)	A5
Baf_ACA-1	-	3 (2)	A1
Baf_PKo	ST71	1 (2)	A2
Baf_HLJ01	ST106	-	-
Bbu_B31	ST1	9 (1)	B4

MLST typing, according to the system described by Margos *et al*.[54] comprising 592 defined profiles, assigned K78 to sequence type ST335 which is identical to the Italian strains 0600839I and 05001891I in the Borrelia MLST database [55]. No match for ACA-1 and Tom3107 was found in the MLST data base with their respective sequence profiles. Column ospA lists the ospA sequence type from the MLSA database [56] and in parentheses the OspA serotypes. The nearest hit for Tom3107 is ospA sequence type-3 with 1 bp mismatch: ospC classification follows the scheme of Seinost *et al*. [57] and Lagal *et al*. [58]. Tom3107 do not fall into any invasive group. For strain HLJ01 only the chromosome sequence is available.

Furthermore, nucleotide sequence data for *ospC* were collected from public databases at NCBI. Initial sequence alignments were prepared with ClustalW [59] and MAFFT [60] sequence alignment software, followed by further manual refinement of the alignment and evaluation of neighbor-joining trees using Jalview [61]. For the figures, the names have been abbreviated, and contain a geographic origin code (international car-code) followed by the strain information (strain name where available, otherwise the accession number or the isolate), and by “H” to indicate human infectious or “Hinv” for human invasive strains. For the partial *ospC* sequences a maximum likelihood tree (RAxML [62]) and a distance tree with split network analysis (SplitsTree4 [63]) were generated.

### Identification of plasmids

The plasmids of K78 (Table 2) have been identified and named based on homologs to the gene encoding the plasmid partitioning protein A (*parA*), which is characteristic for the plasmid compatibility type and sets up the paralogous family (PFam) 32 (S2 Table) as suggested earlier [9, 46, 64].

**Table 2:** Comparison of the replicons found in *Borrelia afzelii* K78 to the published sequences of *B*. *afzelii* strains ACA-1, PKo and *B*. *burgdorferi* strain B31.

		*B*. *afzelii* K78	*B*. *afzelii* ACA-1	*B*. *afzelii* PKo	*B*. *burgdorferi* B31
**Plasmids**	circular	5	5	8	9^e^
**Plasmids**	linear	8	9	9	12

Replicon^a^	Code	Length [kbp]	GC%	Length [kbp]	GC%	Length [kbp]	GC%	Length [kbp]	GC%
**Chromosome**	-	905.9	28.3	903.5	28.2	903.6^d^	28.3	910.7	28.6
**cp9** ^b^	C							9.4	23.7
**cp26**	B	26.6	25.9	26.5	25.8	26.5	25.9	26.5	26.3
**cp32–1**	P			31.5	29.2	30	29.2	30.8	29.4
**cp32–3**	S	30.8	29.2	31	28.9	31.1	29.3	30.2	28.9
**cp32–4**	R	30.5	29.1	30.8	28.8			30.3	29.3
**cp32–5**	V	30.4	29.2	31.2	29.1	30.9	29.2		
**cp32–6**	M							29.8	29.3
**cp32–7**	O					25.3	28.5	30.8	29.1
**cp32–8**	L							30.9	29.1
**cp32–9**	N	31.3	28.7			29.4	19.2	30.7	29.3
**cp32–11**	W					30.7	28.9		
**cp32–12**	X					30.1	29.2		
**lp5**	T							5.2	23.8
**lp17**	D	25.2	24.2	26	23.7	24.7	24	16.8	23.1
**lp21**	U							18.8	20.6
**lp25**	E							24.2	23.4
**lp28–1**	F	28.3	31.9	28.8	31.9			28.2	32.7
**lp28–2**	G	28.7	24.4	32	26.8	26.6	24.2	29.8	31.6
**lp28–3**	H	24.3	24.4	23.3	24.3	22.9	24.4	28.6	25
**lp28–4**	I	25.2	24.1	30.3	24.2	22.4	23.4	27.3	24.5
**lp28–7**	AA			33.4	31	27.2	30.9		
**lp28–8**	AC	28.6	30.8			20.7	29.6		
**lp32–10 (lp56)**	Q^c^			31.3	25.6	30.2	24.8	50	27.3
**lp36**	K							36.8	26.9
**lp38**	J	36.1	23.5	33.5	23.3	33.9	23.5	38.8	26.1
**lp54**	A	57.2	27	60.7	27	57.9	27	53.7	28.1

^a^ Accession numbers (GenBank, RefSeq) are listed in S3 Table.

^b^ Another cp9 plasmid has been described for B31 which is named cp9–2 (renaming the listed to cp9–1) [65]

^c^ The attribution to code “Q” which is the naming for cp32–10 has been made via the presence of the respective plasmid partitioning protein type of the paralogous family 32 (PFam32). The linear plasmid lp56 in B31 is longer and contains parts analog to the cp32–10 type plasmids therefore this plasmid has been proposed to be attributed to code “Q” [46]. Linear plasmids lp32–10, as seen in PKo and ACA-1, carry a PFam32 gene similar to cp32–10 and therefore also get the code “Q” in spite of carrying different gene content.

^d^ There is data from an earlier PKo genome project available, with a chromosome length of 905.4 kbp, GenBank CP000395) with an apparent insert of two genes (BAPKO_0393, BAPKO_0395) and a full definition of the 3’-terminal *arcB* gene (truncated in the listed chromosome).

^e^ Two more plasmids, cp32–2, which has identical PFam32 and PFam49 genes as cp32–7, and cp32–5 have been described in [66] but have not yet been sequenced in full length.

### Nucleotide sequence accession numbers

The fully annotated sequences have been deposited in GenBank and are available under the accession numbers (S3 Table): Chromosome: CP009058, cp26: CP009060, cp32–3: CP009070, cp32–4: CP009069, cp32–5: CP009071, cp32–9: CP009068, lp17: CP009061, lp28–1: CP009062, lp28–2: CP009063, lp28–3: CP009064, lp28–4: CP009065, lp28–8: CP009066, lp38: CP009067, lp54: CP009059.

GenBank accessions and BioProject numbers (NCBI) of the sequences in this publication are: *B*. *afzelii* strains K78: this work (PRJNA158661); PKo: CP002933 (PRJNA159867/PRJNA68149) and PKo: CP000395 (PRJNA58653/PRJNA17057); ACA-1: ABCU02000001–2 (the chromosome sequence is still in draft status and available in the form of two contigs, PRJNA54821/PRJNA19841); HLJ01: CP003882 (PRJNA177930/PRJNA176667); Tom3107: CP009212 (PRJNA218503); *B*. *burgdorferi* strain B31: AE000783 (PRJNA57581/PRJNA3); *B*. *garinii* strains PBr: contigs ABJV02000001–4 (902096 bp, incomplete draft sequences, contig 5 left out from alignment, PRJNA55059/PRJNA28625), BgVir: CP003151 (905534 bp, PRJNA162165/PRJNA72847), NMJW1: CP003866 (902789 bp, PRJNA177081/PRJNA175615), *B*. *bavariensis* strain PBi: CP000013 (high passage (300x), 904246 bp, PRJNA58125/PRJNA12554).

## Results and Discussion

### The genome organization of *Borrelia afzelii* strain K78 resembles other *B*. *afzelii* genomes

The high prevalence of *Borrelia afzelii* in Lyme borreliosis (LB) cases in Europe stresses the importance for establishing a larger genomic database for this species to gain a better understanding of its pathogenicity. For this reason we have sequenced and annotated the whole genome of the *B*. *afzelii* strain K78, which has been isolated from a human LB lesion (primary erythema migrans).

### Characterization of the K78 linear chromosome

The sequence of the linear chromosome was mainly obtained by Sanger shotgun sequencing. The K78 chromosome consists of 905,949 nucleotides and its length matches those of the chromosomes of other sequenced *B*. *afzelii* strains, PKo (erythema migrans, Germany) [11, 18], HLJ01 (blood, China) [19] Tom3107 (*Ixodes persulcatus*, Russia) [20] and ACA-1 (acrodermatitis chronica atrophicans, Sweden) [11] which are in the range of 903,516–905,861 bp. The major difference in length of the *B*. *afzelii* chromosomes is caused by sequences located at their 3’-ends. All sequenced *B*. *afzelii* chromosomes show an overall G+C content of 28.3% which is close to the value for *B*. *burgdorferi* B31 which is 28.6% (Table 3).

**Table 3:** Comparison of the K78 chromosome to representative chromosomes within Borrelia.

Organism	Length bp	GC%	Identity % ^a^ (to K78)	Indel content % ^a^ (to K78)
*B*. *afzelii* K78	905,949	28.3	100 (ref)	0 (ref)
*B*. *afzelii* ACA-1	>903,516^b^	28.3*	99.4*	0.3*
*B*. *afzelii* PKo	903,609	28.3	99.5	0.3
*B*. *afzelii* HLJ01	905,471	28.3	99.4	0.1
*B*. *afzelii* Tom3107	905,861	28.3	99.4	0.1
*B*. *burgdorferi* B31	910,724	28.6	91.1	1.7
*B*. *bavariensis* PBi	904,246	28.3	92.7	0.8
*B*. *garinii* PBr	>902,096^c^	28.3*	92.4*	1.1*
*B*. *garinii* Vir	905,534	28.4	92.9	0.7
*B*. *garinii* NMJW1	902,789	28.4	92.6	1.0

^a^Sequence identities and indel contents calculated with stretcher (EMBOSS package [44])

^b^Sum of two unconnected non-overlapping contigs (436,767 + 466,749 bp)

^c^Unfinished assembly (5 contigs, of which the shortest with 1774 bp length has been left out of the comparative analysis)

*Approximate values due to incompleteness of the chromosome assemblies.

A comparison of the *B*. *afzelii* K78 chromosome with PKo, ACA-1, HLJ01 and Tom3107 by pairwise global alignment shows an extremely close relationship with sequence identities above 99.4%, whereas a sequence identity of 91.1% is seen for *B*. *burgdorferi* B31, in agreement with a previous study [18]. However, higher sequence conservation than to *B*. *burgdorferi* is observed between K78 and *B*. *garinii* strains (PBr, Vir, NMJW1 [92.4–92.9%]) and *B*. *bavariensis* (PBi [92.7%]). The amount of indels in *B*. *afzelii* is 0.1–0.3% which in non-*B*. *afzelii* chromosomes are higher, 0.8–1.7% (Table 3, S1 Fig.). The evolutionary stability of the linear chromosomes of different *Borrelia* species indicates that adaptions resulting in immune evasion and host specificity and human disease patterns took and take place on the various plasmids rather than the chromosome.

### The 3’-end of *B*. *afzelii* chromosomes

A high similarity between the *B*. *burgdorferi* chromosomes has been described previously [67]. However, as an exception some variability was observed at the 3'-end of chromosomes arising from extensions with sequences derived from different plasmids [46, 68]. These kind of exchange processes with plasmids at the chromosomal 3'-end have not been reported for the published *B*. *afzelii* genomes (ACA-1, PKo, HLJ01 and Tom3107), which is also not the case for the K78 chromosome. However, the complete 3’-end of a *B*. *afzelii* chromosome has only been reported for the strain R-IP3 [68]. The 3’-ends of K78 and R-IP3 (GenBank accession AF008219) after the stop codons of their last open reading frames including the telomeres are 209 and 271 bp long, respectively. The non-coding 3’-ends match over a region of 109 bp. In contrast, the 3’-end of the *B*. *burgdorferi* chromosomes are different in length and in sequence [46, 68]. Thus, it seems that the linear chromosomes of *B*. *burgdorferi* have undergone recombination with one or several linear plasmids after the evolutionary separation from *B*. *afzelii*.

### Locations of variable regions and non-coding RNAs of the *B*. *afzelii* chromosome

A closer look at the chromosome sequences of the six strains, K78, ACA-1, PKo, HLJ01, Tom3107 and B31, showed a consistent homology and synteny over the complete chromosome. There are only few positions with elevated variability. To be mentioned in this respect are the sites coding for proteins with a variable number of tandem repeats like *lmp1*, *infB* and BB_0546 (BAFK78_546), and the locus with the ribosomal RNAs 16S (*rrs*), 23S (*rrl*) and 5S (*rrf*) which includes the variable intergenic spacer regions *rrs-rrlA* and *rrfA*-*rrlB*. The gene corresponding to BB_0524 (BAFK78_0522) is conserved among the five *B*. *afzelii* strains and has been described as unusually variable with a high number of indels, the difference in indels has been proposed for differentiation between *Borrelia* species [67].

**Fig 1:**
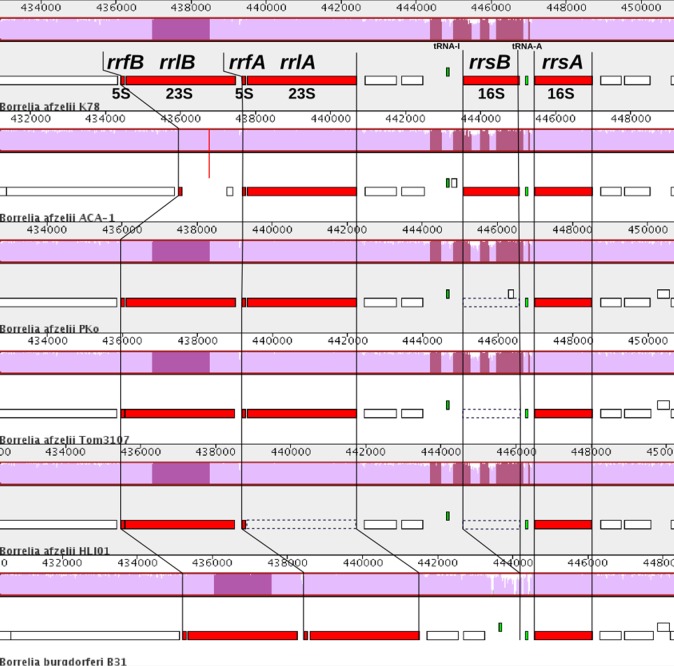
Chromosomal region of 5S-23S rRNA and 16S rRNA for the *B*. *afzelii* strains K78, ACA-1, PKo, Tom3107, HLJ01 and for *B*. *burgdorferi* B31. The rRNAs (marked red), are presented with transcription from right to left as located on the chromosome, and are composed of two copies of 16S rRNA, separated by tRNA-Ala. A tRNA-Ile (transcribed left to right) precedes the tandem repeats of the 23S-5S cassette. In many cases one of the 16S copies has undergone degeneration. In the case of ACA-1 the two contigs constituting the chromosome are separated at the position where the second 23S rRNA copy is expected (vertical red line), meaning the presence or absence of the second copy of 23S rRNA could not be determined due to the lower sequencing quality in this region. There is a high sequence homology among the four *B*. *afzelii* strains (except for the second copy of 23S rRNA of ACA-1) in contrast to the sequences in *B*. *burgdorferi* B31 rRNAs. The similarity score plots of the Mauve alignments use the backbone color scheme [43] which shows overall similarity in a mauve color or clustering blocks among cluster members in the same color.

The K78 genome encodes 33 tRNAs covering all 20 natural amino acids. Eleven additional loci comprising non-coding RNAs have been identified, six of them encode ribosomal RNAs (rRNA). A comparison of the rDNA loci (Fig. 1) showed that the 23S-5S rDNA (transcribed from the opposite strand) are present in tandem repeats (*rrlA*-*rrfA* and *rrlB*-*rrfB*). However, the *rrlA* locus in HLJ01 has not been annotated. The gap between the two chromosomal contigs of ACA-1 is at the position of a potential tandem repeat of 23S-5S rDNA, between *rrfA* and *rrfB* (Fig. 1). The first 23S-5S rDNA repeat is preceded by one (*B*. *burgdorferi*) or two (*B*. *afzelii* and *B*. *garinii*) heterogeneous copies of 16S rDNA, *rrsA* and *rrsB* [69]. The latter appears to be a pseudogene and is not annotated in HLJ01, Tom3107 and PKo. Compared to the 23S-5S rDNA tandem repeats which are highly conserved (>99%), there is a relatively low sequence identity (77–79%) between the 16S rDNA repeats (intra-species). The *rrsB* genes from the strains *B*. *afzelii* K78, PKo, ACA-1, HLJ01 and Tom3107 have a slightly lower sequence identity (95–99%) compared to *rrsA* (>99%). A large scale comparison of prokaryotic 16S rDNA genes and their substitution patterns [70] together with its lower GC content (~38% vs ~47%) compared to *rrsA*, support the hypothesis that *rrsB*, might be a nonfunctional rDNA gene in *B*. *afzelii*.

Five other non-coding RNA sequences have been identified in the K78 genome, the RNA subunit of RNase P (*rnpB*) (Rfam family RF00010), the small signal recognition particle RNA (*ffs*) (RF00169), a transfer-messenger RNA (tmRNA) (RF00023) and analogs to *dsrA* and *ssrS* (6S RNA). In *B*. *burgdorferi*, *dsrA* has been shown to be involved in translational regulation of RpoS [71], *ssrS* binds to the RNA polymerase holoenzyme and regulates gene expression at the shift from exponential growth to stationary phase [72]. The genes *ffs*, *dsrA* and *ssrS* have previously not been annotated in any of the complete genomes of the different *Borrelia* species.

### Functional classification of ORFs

The genome of *B*. *afzelii* K78 has been characterized with a combination of automated annotation followed by manual curation and correction. A classification of the proteins into functional categories as defined by NCBI with clusters of orthologous groups (COG) is summarized in Table 4 (S2 Fig., S3 Fig.). Approximately 79% of the chromosomal proteins but only 14% of the plasmid encoded proteins could be attributed to a COG (rpsblast, E-value cut-off 0.01), resulting in a total assignment of 54% of the annotated genome. By manual curation and inclusion of results from the conserved domains database (CDD), the assignment to a cluster or group could be increased to 58%.

**Table 4:** Functional classification of the *B*. *afzelii* K78 annotated genome, describing a total of 1,309 proteins.

Chromosome (n = 813)	Plasmids (n = 496)	Functional category (COG)
27	4	Amino acid transport and metabolism
49	4	Carbohydrate transport and metabolism
14	14	Cell division and chromosome partitioning
54	1	Cell envelope biogenesis, outer membrane
52	0	Cell motility and secretion
12	1	Coenzyme metabolism
9	4	Defense mechanisms
51	7	DNA replication, recombination, and repair
22	1	Energy production and conversion
66	8	General function prediction only
22	1	Inorganic ion transport and metabolism
32	0	Intracellular trafficking and secretion
15	0	Lipid metabolism
20	7	Nucleotide transport and metabolism
32	0	Posttranslational modification, protein turnover, chaperones
1	1	Secondary metabolites biosynthesis, transport, and catabolism
30	0	Signal transduction mechanisms
23	2	Transcription
118	0	Translation, ribosomal structure and biogenesis
42	12	Function unknown
173	429	Unclassified in COG

The best-hits per category from rpsblast against COG with a cutoff of E-value 0.01 are counted. Proteins with the best-hit falling into more than one category are counted as hit in each category which results in the addition of 51 hits, resulting in a total of 758 hits to defined COGs.

Of the 496 proteins encoded on plasmids, a relatively high number [85] of open reading frames apparently are no longer under selective pressure and seem to be in a state of degradation, have damaged reading frames (truncated, genuine frameshifts) or are undergoing duplications and rearrangements as previously described for *B*. *burgdorferi* [9].

*Borrelia* genomes contain an exceptional high number of lipoproteins. By using SpLip, 105 lipoproteins were predicted for K78 and more than 70% of those genes are located on plasmids (Table 5). This finding resembles the situation described for *B*. *burgdorferi* strain B31, where 105 lipoproteins have been predicted of which 60% are located on plasmids [8]. However, the notion is that the number of lipoproteins is underestimated [9]. Thus, the true number of lipoproteins present in K78 may even be higher than 105.

**Table 5:** Number of predicted membrane proteins in four *B*. *afzelii* strains and *B*. *burgdorferi* B31.

Genomes	Lipoproteins^a^		Signal peptides^b^		Transmembrane helices^c^	
	Chromosome	Plasmids	Chromosome	Plasmids	Chromosome	Plasmids
Baf_K78	31	74	98	30	191	48
Baf_ACA-1	28	72	91	40	190	48
Baf_PKo	31	85	89	38	192	53
Baf_HLJ01	27	-	89	-	197	-
Bbu_B31	36	74	87	42	179	58

^a^Lipoprotein predictions (SpLip). Given counts are “probable” and “possible” hits combined.

^b^Signal peptide prediction (SignalP) were not counted when SpLip predicted a lipidation signal for the protein.

^c^The predictions of a single transmembrane helix (TMHMM) was not counted as such when located within the N-terminal 60 amino acids and SignalP predicted a signal protein or SpLip a lipidation site.

### Sequence typing of the *Borrelia afzelii* genomes classifies K78 to a cluster with invasive strains

**Fig 2:**
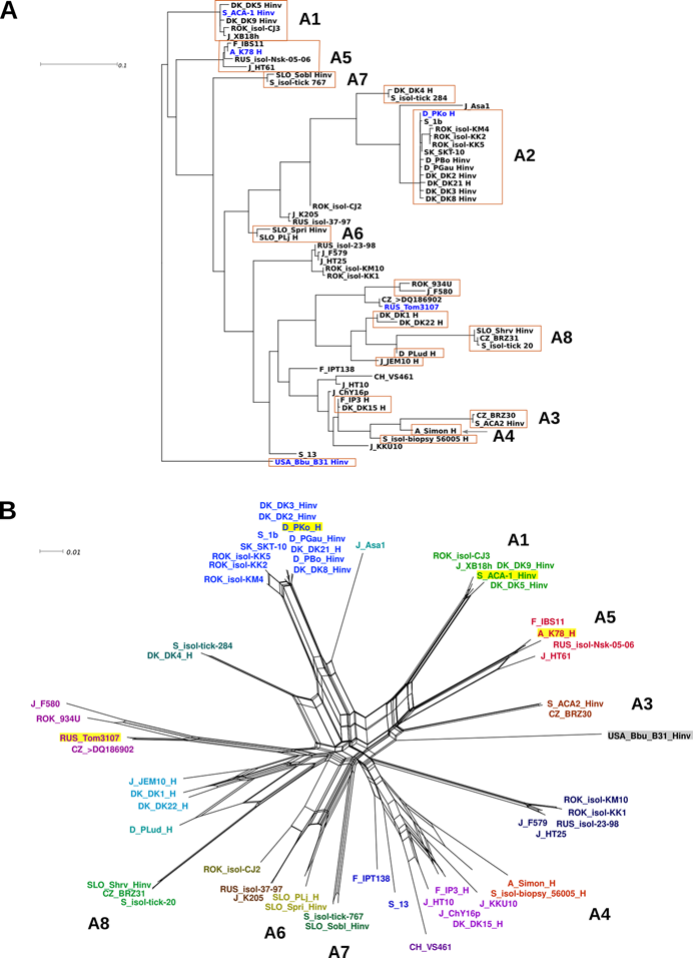
Alignment of *B*. *afzelii* K78 *ospC* sequence against the sequences of *B*. *afzelii* strains from public databases. A non-redundant set of partial *ospC* sequences according to BAFK78_B0019 bp 97–583, comprising 59 *B*. *afzelii* strains and the sequence of *B*. *burgdorferi* B31 as external root reference were included in the analyses. A: Maximum likelihood tree representation, re-rooted with *B*. *burgdorferi* B31 as outgroup. Clusters containing strains attributed to human infectivity are boxed, of which the previously identified groups were labelled A1–A8. The strains compared in this study are highlighted in blue. B: A recombination network representation is shown for the sequences in an unrooted distance phylogram. The pairwise homoplasy index test for the *B*. *afzelii* sequences (p = 7.8x10^-15^) indicates significance for the presence of recombination events. The strains compared in this study are highlighted by a yellow background.

The classification of *Borrelia* strains as defined by Lagal *et al*. [58] makes use of the genetic variability of the *ospC* gene. To evaluate the relationship of the four *B*. *afzelii* strains discussed here with a total of 59 known non-redundant *B*. *afzelii ospC* sequences (S3 Table), a central fragment of 442 to 460 bp with high variability [58] was aligned. *B*. *burgdorferi* B31 was included as an out-group to generate a split network analysis and a maximum-likelihood tree (Fig. 2A-B). Split networks help to visualize reticulate events like recombination, hybridization, reassortment or horizontal gene transfer and the indicated edge lengths are proportional to the weight (degree of reticulate events) of the associated splits [63]. Significant evidence of recombination could be found for the 59 *B*. *afzelii ospC* sequences (p = 7.8x10^-15^) by the pairwise homoplasy index test [73] as has been shown for *B*. *burgdorferi* [74]. The nomenclature, A1–A8, in Fig. 2A-B designates *ospC* clusters according to the scheme and assignment by Seinost *et al*. [57] and Lagal *et al*. [58]. The *ospC* clusters include human isolates defined as either invasive (isolated from disseminated infection, e.g. CSF, blood, multiple erythema migrans) or non-invasive (isolated from localized infection, e.g. primary erythema migrans) depending on the source of isolation. K78, a non-invasive strain (isolated from a primary erythema migrans) is found in cluster A5, ACA-1 is found in cluster A1, and PKo in cluster A2 (Fig. 2B). Thus, all three strains belong to clusters containing invasive strains. Tom3107 which is a tick isolate is not found in any of the clusters with human isolates. Worth to mention is that two distinct clusters are only made up of strains isolated in Asia (South-Korea, Japan and Russia) and one of these clusters contains a human isolate. Another distinct cluster is made up of two strains isolated in Slovenia, suggesting a local geographical distribution of certain *ospC* types. Thus, there does not seem to be a clear correlation between *ospC* type and pathogenesis in humans, since most clusters do contain strains isolated from humans both invasive and non-invasive.

A multilocus sequence typing (MLST) scheme based on eight chromosomal housekeeping genes of *B*. *burgdorferi* has been defined to better understand the dynamics of the epizootic spread and to predict the evolutionary trajectories of *B*. *burgdorferi* [54]. This scheme has the advantage that the influence of plasmid loss, inter-plasmid gene exchange and degradation processes, especially observed for the linear plasmids, has no influence on the classification. MLST with 592 defined allelic profiles from “borrelia.mlst.net” [55] shows that the five *B*. *afzelii* strains (K78, ACA-1, PKo, Tom3107 and HLJ01) belong to different sequence types (Table 1). A “population snapshot” with eBurst3 (http://eburst.mlst.net) and a neighbor-joining tree created with Jalview confirmed that the *B*. *afzelii* genomes are sufficiently distinct to be members of different MLST main clusters.

### Plasmid composition of *B*. *afzelii*

*Borrelia* genomes are complex due to the presence of a large number of both linear and circular plasmids which represent about 30% of the genomic information. The situation is even further complicated by the fact that plasmids not essential for *in vitro* cultivation can be lost after multiple passaging [75, 76]. A high-throughput analysis of the plasmid content in *B*. *burgdorferi* B31 has revealed loss of the plasmids lp5, lp56, lp28–1, lp25, cp9, lp28–4, lp28–2 and lp21 (in the order of decreasing frequency) during *in vitro* cultivation [77]. Others observed that the plasmids most frequently lost were lp5, cp9, lp21, lp28–1 and cp32–6 [78]. It has also been described that plasmids which are essential for the passage in ticks, bird or mammals, may not be essential for *in vitro* cultivation [79, 80], as for example lp28–1 in *B*. *burgdorferi* B31 (lp28–8 in *B*. *afzelii* K78) which harbors the variable major protein-like sequence E (VlsE) surface antigen essential for efficient immune escape in the host [81]. Therefore, the number of identified plasmids may be underestimated in any of the reported genomes. In the K78 genome, 8 linear and 5 circular plasmids have been identified, PKo was reported to possess 9 linear and 8 circular plasmids and ACA-1 9 linear and 5 circular plasmids (Table 2). These numbers indicate fewer plasmids in *B*. *afzelii* compared to B31 with 12 linear and 9 circular plasmids. There are five paralogous families (PFams) associated with plasmid maintenance and consisting of putative replication and plasmid partition genes [9], PFams 32, 49, 50, 57 and 62, of which PFam32 (*parA*), has been used to identify and name the plasmids in this study. The presence of the ParA plasmid partitioning proteins allows the assignment to the orthologous replicons, which is also reflected in the analog naming of the plasmids across organisms. A comparison of the variation in plasmid composition shows a relatively homogeneous composition among the *B*. *afzelii* strains K78, PKo and ACA-1, but reveals a number of significant differences to *B*. *burgdorferi* B31 (Table 2). The main difference in plasmid content of the *B*. *afzelii* strains lies in the number of plasmids belonging to the cp32 and lp28 families which are very redundant in their gene content.

### Pathogenicity related genes and their presence on plasmids reveal gene shuffling

Plasmids lp17, lp38, lp54, cp26 and a varying number of cp32 and lp28 can be found in all *B*. *afzelii* strains (Table 2, S4 Fig., S5 Fig.), whereas plasmids like cp9 and the linear plasmids lp5, lp21 lp25 and lp36 of B31 have no counterparts in the *B*. *afzelii* genomes (Table 2). It can be speculated that if homologous plasmids exist in *B*. *afzelii* they must have been lost during *in vitro* cultivation or that required virulence genes are located on other plasmids.

### Virulence genes from *B*. *burgdorferi* B31 lp25 and their location on linear plasmids of *B*. *afzelii*

Loss of lp25 in *B*. *burgdorferi* has been associated with reduced colonization of the tick gut [82] and with decreased infectivity of mice [83]. One of the virulence genes on lp25 is *pncA* (BB_E22) which is needed for infectivity of mice [84] is found in K78 and ACA-1 on plasmid lp28–2, which is only partially related to *B*. *burgdorferi* lp25. Another virulence gene on lp25 is *bptA* (PFam99, BB_E16) which is essential for the persistence of *B*. *burgdorferi* in ticks [85], the homolog in *B*. *afzelii* K78, ACA-1, and PKo is also located on lp28–2. The gene *bbe31* encodes a virulence-associated lipoprotein (PFam60), which promotes migration of spirochetes in ticks from the midgut to the salivary glands [86]. The PFam60 family has multiple members, which are found on a variety of linear plasmids (*B*. *burgdorferi* B31: lp25, lp28–3, lp28–4, lp36 and lp56, and in *B*. *afzelii* K78, ACA-1, PKo: lp17, lp28–2,-3,-4, lp38 and, lp54) and it is likely that one of the PFam60 proteins located on a different plasmid of *B*. *afzelii* can substitute for the function of BB_E31 in *B*. *burgdorferi*.

### Virulence genes from *B*. *burgdorferi* B31 lp36 and their location on linear plasmids of *B*. *afzelii*

Plasmid lp36 in *B*. *burgdorferi* B31 is not needed for *in vitro* cultivation or survival in the tick but is needed for infectivity in mammals [87]. Adenine deaminase (PFam61, BB_K17) which is located on lp36 is needed for the infectivity in mammals [78, 87]. The *B*. *afzelii* strains K78, PKo and ACA-1 are all lacking a homolog to plasmid lp36, but the *adeC* gene homolog is located on plasmid lp38 in these strains. The region *bbk02*.*1*-*bbk04* on lp36 consists of short overlapping genes in B31, but appears to contain a longer open reading frame in other *B*. *burgdorferi* strains. Frame-shifts in genes of some *B*. *burgdorferi* strains and insertion of a transposon in some strains cause a reduction in infectivity [78]. In both *B*. *afzelii* strains K78 (lp28–1, BAFK78_F001) and ACA-1 (lp28–7, BafACA1_AA34), the region corresponding to *bbk02*.*1*-*bbk04* is annotated as one longer open reading frame, predicted to be a type I restriction enzyme. It has been demonstrated for *B*. *burgdorferi* B31 that spirochetes lacking the gene *bbk46* (Pfam75), encoding a putative immunogenic lipoprotein (P37), are not able to maintain a persistent infection in mice [88]. Homologs of PFam75 in *B*. *afzelii* are found on lp32–10 in ACA-1 and PKo and on lp28–8 in K78, although with apparent frame-shifts. However, they have relatively low sequence identities to BB_K46 (32–40%). This low sequence identity might indicate that the homologs in *B*. *afzelii* have a different function and/or are not essential for a persistent infection.

The protein BB_K32 on lp36 in *B*. *burgdorferi* B31 is a virulence factor, which binds to fibronectin [89–91] and promotes binding to glycosaminoglycans (GAG) in a similar manner as decorin binding protein A (DbpA) and B (DdbB) as well as Bgp [90]. In spite of the role of BB_K32 in pathogenicity a *bbk32* deletion mutant of *B*. *burgdorferi* B31 has been shown to be fully infective in mice [92], indicating that other GAG-binding adhesion factors can at least partially compensate for the loss of BB_K32 [92]. In contrast to *B*. *burgdorferi* B31, the homolog of *bbk32* is located on plasmid lp17 in *B*. *afzelii* strains (ACA-1, K78 and PKo).

### Sequence variation of the conserved lp54 plasmid is mainly due to genes involved in host adaption

Many genes located on the conserved lp54 plasmid have low sequence identity across *Borrelia* species, among them are the genes encoding decorin binding proteins A and B (*dbpA* and *dbpB*, PFam74) and the complement regulator-acquiring surface protein 1, CRASP-1, paralogous family 54 (PFam54). The surface lipoproteins DbpA/DbpB bind to the proteoglycan decorin [93, 94]. While DbpB shows high conservation within species (99–100%) and across species (>65%), the more variable DbpA shows sequence identities of >81% within *B*. *afzelii* and only 40–45% between the three *B*. *afzelii* strains and *B*. *burgdorferi* B31, in agreement with the reported species specific grouping of *Borrelia* species [95]. Residues of the basic surface patch of DbpA, which represents the putative GAG binding site, are only partially conserved in *B*. *afzelii*. The DbpA sequence of K78 shows notable differences to ACA-1 and PKo (81–82% sequence identity) while the latter two are more similar (92% sequence identity). The differences also include residues of the basic patch, which may have an effect on the tissue tropism in the host. Both, DbpA and DpbB have been proposed for serodiagnostic applications [96]. PFam54 encompasses many genes which are grouped together in a tandem array on lp54. The tandem array in *B*. *burgdorferi* B31 (BB_A64-BB_A73) contains several surface-localized proteins which are expressed during persistent infection of immune competent mice [97]. Variability in gene content by duplication/deletion and gene diversification has been described especially for the region located between the genes analog to *bba66* and *bba73* in a set of 10 *Borrelia* lp54 plasmids [98]. The highest number of genes in the PFam54 tandem array is found in *B*. *afzelii* PKo. PKo appears to have one gene copy (BafPKo_A0065) inserted when compared to K78 and ACA-1.

### Telomeres of linear genetic elements in K78 can be assigned to three known telomere types

**Fig 3:**
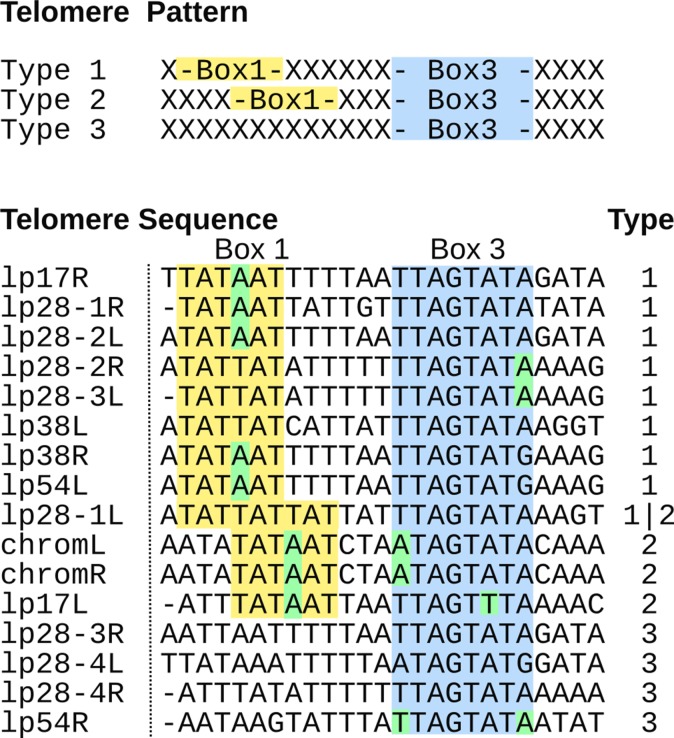
Telomere types of the linear replicons. Alignment of the telomeres of the linear replicons in *B*. *afzelii* K78 is shown. The sequences are oriented such that their hairpin bend would be positioned to their left side. The typing corresponds to the classification of the telomere types 1–3 according to the spacing between Box 1 (yellow) and Box 3 (blue) or the absence of Box 1 [68, 99, 101]. For five of the sequences the utmost left residue could not be determined and is represented by a “-”as placeholder. Box 1 and Box 3 correspond to previously annotated regions of conservation which are assumed to be directly (Box 3) or indirectly (Box 1) involved in interaction with the telomere resolvase ResT [100, 102]. No telomere data could be obtained for K78 lp28 and the telomeres of lp54L and lp38R are identical. In Box 1 two different sequences, TAT(A/T)AT, are present as in B31. Unlike in B31, where the TATTAT sequence is exclusively found in type 2 telomeres, this sequence is also found in type 1 telomeres of K78. lp28–1L of K78 is an exception while it is compatible with both the definition of type 1 and type 2 telomeres as also seen on lp28–3R of B31 and lp28–2R of PKo. Within the 16 telomeres, 6 have substitutions in Box 3 (5 with one change, and 1 with two changes, marked green).

The genomes of *Borrelia* species contain multiple linear replicons, which are flanked with short telomere structures forming covalently closed hairpins. Both ends of the linear chromosome in K78 contain a type 2 telomere with a 34 bp reversed repeat [99, 100], similar to the telomeres of the *B*. *afzelii* strain R-IP3 [68]. Most of the linear K78 plasmids could be completely sequenced including their telomeres and assigned to the three different telomere types (Fig. 3). All three telomere types are represented in the K78 genome. Most of the linear plasmids have different telomere types at both ends, which might be a result of frequent recombination seen with the linear plasmids. The telomere of the left end of lp28–1 has the features of both type 1 and 2 telomeres, which is also seen in right end telomeres of lp28–2 in PKo and lp28–3 in B31.

### A species specific variable region of circular plasmid cp26

**Fig 4:**
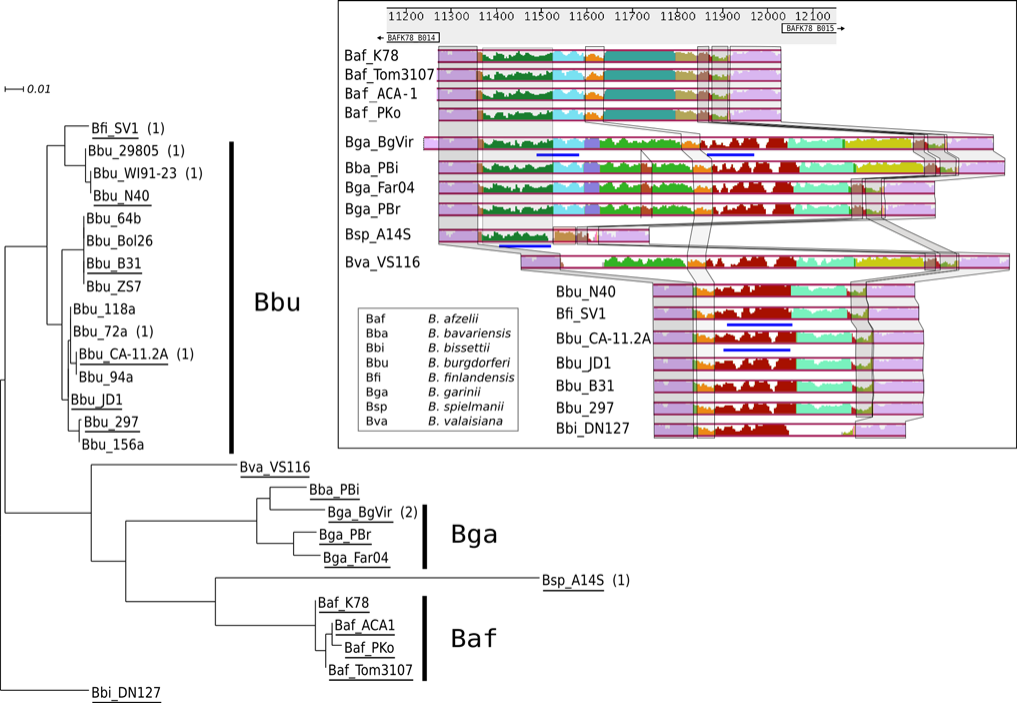
Species-specific variation of the intergenic variable region in the circular plasmid cp26. The variable sequence segment in the cp26 plasmids of K78 is compared with 25 *Borrelia* strains. A maximum likelihood tree rooted with a *B*. *bissettii* sequence as outgroup, shows the relationship for the intergenic part, which in *B*. *afzelii* K78 is situated between BAFK78_A0014 and BAFK78_A0015 (bacterial extracellular solute-binding protein), and which shows a species-specific length. Insertions and deletions within this region have been analyzed with the program Mauve, and the compositional analysis for 16 of the 26 sequences (underlined in the tree view) is shown with related segments marked by color and/or boxes together with a similarity score diagram for each sequence. Blue bars denote segments which, in some strains, have been annotated as short hypothetical proteins (the number of assigned proteins in this region is indicated in parentheses in the tree view).

The circular plasmid cp26 is essential for viability and the gene content and synteny is highly conserved between species. Besides the lower conservation of *ospC*, a certain degree of sequence variability is seen in several intergenic regions, most notably in a region upstream of *oppAIV* (coding for an extracellular solute-binding protein of an oligopeptide ABC transporter). In K78 this region is located between BAFK78_B014 and BAFK78_B015. The observed insertions and deletions appear to be species specific to a high degree when compared to a set of 26 cp26 plasmids from different strains (Fig. 4, S3 Table). Even though multiple insertions and deletions occur at this location, the overall gene structure of the cp26 plasmids is highly conserved across *Borrelia* species, underlining the high importance of cp26. The longest inserts and highest number of inserted sequence elements are found in *B*. *garinii*, in some strains 1 or 2 short (<50 amino acids) hypothetical open reading frames have been annotated. This intergenic region could therefore probably be a useful target for diagnostic identification of *Borrelia* species, considering the importance for *Borrelia* to maintain the cp26 plasmid.

### The circular plasmid family cp32 in K78

A varying number of circular plasmids with comparable size, named cp32, are present in all *Borrelia* genomes. These plasmids show high sequence conservation between different strains and contain essential genes for virulence. A comparison of the four different cp32 plasmids of K78 underlines their generally high sequence conservation, since there are only indels at few positions. One position contains two adjacent genes, encoding the PFam32 and PFam49 plasmid partitioning proteins and a PFam80 family protein (Bdr), another position harbors the highly diverse *erp*-locus (which codes for outer surface proteins of PFam163 (OspE/OspF/Ebf)) and an additional position the *mlp*-locus (PFam113). The *mlp*-locus contains a Bdr-like KID repeat family protein (PFam80), except on cp32–9, where instead a Rev family protein (PFam63, BAFK78_N027) is located on the opposite strand. RevA in *B*. *burgdorferi* (BB_M27, BB_P27, BBC10) is known to bind mammalian fibronectin, as BB_K32, [103] and is required for infectivity in mice [78]. The conservation pattern in K78 is consistent with what has been described for cp32 plasmids from *B*. *burgdorferi* B31 and 297 [104]. In summary, the high evolutionary stability of the cp32 plasmids across the *Borrelia* species underlines their importance for bacterial survival.

### Presence of prophage DNA in cp32 plasmids

The cp32 plasmids were first identified as prophage genomes in *B*. *burgdorferi CA-11*.*2A* [105] and four members of the cp32 family have been identified in K78. Among the spirochetes associated with LB, the cp32 prophages can be classified into a scheme of 12 different types according to the respective plasmid partitioning protein, ParA [64]. The definition of 12 types has been verified in an analysis of 22 *Borrelia* strains and it was suggested that they cover the available diversity of cp32 types [106]. In *B*. *burgdorferi* B31 cp32–1, -2, -3, -4, -5, -6, -7, -8, -9 [9, 66] and in *B*. *afzelii* K78 cp32–3, -4, -5, -9 have been identified. ACA-1 has 4 and PKo 7 cp32 plasmids. In B31, lp56 contains an integrated cp32, which makes lp56 bigger than its analogs, lp32–10, in ACA-1 and PKo (no analog to lp56 has been identified in K78). More distantly related cp32-like prophage-type sequences can be found inserted in the linear plasmid lp54 of *B*. *afzelii* (K78, ACA-1, PKo, Tom3107) and similarly in *B*. *burgdorferi* B31, interrupted by insertions and replacements and in lp28 members (K78, ACA-1: lp28–1; B31: lp28–2) (S6 Fig. and [9]).

In *B*. *burgdorferi*, operons with 30 co-transcribed genes have been reported for the circular plasmids cp32–8 (BB_L42 to BB_L43 followed seamlessly by BB_L01 to BB_L28) and cp32–7 (BB_O43 to BB_O44 followed seamlessly by BB_O01 to BB_O28). These operons encode several bacteriophage homologs [107]. Similar operons were found with high conservation and synteny in ACA-1 and PKo. However, in K78, a premature stop codon is present in one (BAFK78_R022) of the late genes on cp32–4, which might have an effect on the stability of the poly-cistronic mRNA and thus the expression of the co-transcribed genes.

In B31, a putative bacteriophage-associated holin (BlyA) has been described [108] as part of a four-gene operon which has been shown to mediate release of latent ClyA cytolysin when expressed in *Escherichia coli* [109]. It is a member of PFam109 which has further members present on each of the cp32 family plasmids and on the linear plasmid lp56 (BB_Q30). In K78, four BlyA holins, BAFK78_N023, BAFK78_R023, BAFK78_S023, and BAFK78_V023, have been identified, and they are situated in operons which are highly syntenic to the B31 counterparts. Another *blyA* family gene, more distantly related, exists on the K78 plasmid lp54, BAFK78_A014 which is similar to BB_A12 in *B*. *burgdorferi* B31.

### Copy numbers of tandem repeats differentiate *B*. *afzelii* strains

In the genomes of prokaryotes a variety of different types of repeats exist. The number of tandem repeats (VNTR), or the multiple-locus variant-repeat analysis (MLVA) are increasingly used as molecular markers since the copy number of repeats often differs between otherwise closely related strains in a characteristic way [110, 111]. The genes on the chromosomes of *Borrelia* species are highly packed and there are only few intragenic spacers, which could harbor extensive repeat content. The genomes of K78, ACA-1, PKo, Tom3107, HLJ01 and B31 were searched for tandem repeats and a list of the most prevalent repeats is available as supplementary material (S4 Table). A repeat has been considered significant, when the total length is above 50 bp with a sequence identity of 80%. Three low-complexity repeat regions were detected within genes of the chromosome, the surface—located membrane protein 1 (*lmp1*, BB_0210), a sporulation/cell division-related hypothetical protein (BB_0546, BAFK78_546) and the translation initiation factor IF-2 (*infB*, BB_0801). These have already been described in B31 [112]. The gene *lmp1* shows a notable variation of the number of tetratricopeptide repeats across the strains with the highest number in K78 [6], when compared to ACA-1 [5], PKo [5], Tom3107 [5], HLJ01 [2] and B31 [2]. Different numbers of short repeats are also seen for *infB* between the strains K78 [10], ACA-1 [12], PKo [6], Tom3107 [10], HLJ01 [10] and B31 [12], and a hypothetical protein (BB_0546, BAFK78_546) for strains K78 [3], ACA-1 [4], PKo [3], Tom3107 [3], HLJ01 [3] and B31 [5]. The combination of the number of repeats of the three loci can uniquely identify these six *Borrelia* strains.

Proteins with KID-repeats (IPR003900) are found on the cp32 and lp28 type plasmids of K78. These repeats contain the tripeptides KID and/or KIE, and are characteristic of the Bdr family (PFam80), which are inner membrane proteins unique to *Borrelia*. Members of this gene family have been described to be environmentally regulated in B31 [113].

The surface-exposed virulent strain associated repetitive antigen (*vraA*, BB_I16) is located on plasmid lp28–4 of B31. It contains a repetitive sequence of 27 bp in 21 copies encoding the invariant motif “EEELKKKQQ”, which is highly polar and responsible for antigenicity [114, 115]. The presence of the plasmid lp28–4 has been related to infectious strains of *B*. *burgdorferi* [114, 115] in which the lipoprotein is highly conserved and only varies in the number of motif repetitions. VraA belongs to PFam60 together with many other members, but which do not carry this repeat motif. There is no direct homolog of VraA in *B*. *afzelii*, but a similar repeat motif of 9 amino-acids, “EEEEKQRQK” is present in *B*. *afzelii Erp* family proteins of PFam163 (BAFK78_H002, BafACA1_H02, BafPKo_H0021), also with varying repeat numbers (14, 7, and 4, respectively).

### Organization of the *vls* silent cassette loci

The membrane lipoprotein VlsE is part of the immune escape mechanisms of *Borrelia*. The diversity of VlsE is generated by recombinational switching using a segmental gene conversion mechanism with a contiguous series of silent cassettes [81]. This allows *Borrelia* to present VlsE with a varying and diverse composition of residues on the cell surface. The *vls* locus of K78 is situated on the linear plasmid lp28–8 and 11 *vls* cassettes have been sequenced (S7 Fig.). With the available sequence data and complementary PCR analyses we were not able to verify the completeness of the *vls* locus because of the highly repetitive sequences. Two cassettes, *vls4* and *vls6*, contain apparently genuine frame-shifts and *vls11* is present as two fragments, representing about half a cassette. The first cassette, *vls1*, is preceded by a sequence similar to the 5’-part of the expression cassette *vlsE* which contains a lipoprotein signal sequence and the N-terminal constant part of VlsE1 which is followed by a silent cassette. A similar architecture with residual *vlsE* sequence at the start of the first cassette can be seen in *B*. *burgdorferi* JD1 lp28–1 (accession NC_017404). However, in JD1 the *vlsE*-analogous part in the first cassette lacks the codon for the N-terminal methionine. JD1 contains the expression cassette *vlsE1* on the opposite strand, similar to the situation in *B*. *garinii* Far04 lp28–1 (accession NC_011873). The first silent *vls*-cassette of B31 contains a partial lipoprotein signal sequence. In *B*. *burgdorferi* B31 the *vlsE1* gene is located adjacent to the *vls*-cassettes, separated by 298 bp with the ORF on the opposite strand (another non-functional copy with frame-shifts is located on plasmid lp38). The *vlsE* expression locus, which is expected to be separated from the silent *vls*-cassettes, was not found in our K78 sequence data set. It can be speculated that it got lost during the course of *in vitro* cultivation before passage 5 which was used for sequencing. This is supported by the observation that plasmids which are important for infectivity, such as lp28–1 in *B*. *burgdorferi* B31 which harbors VlsE, are not essential for *in vitro* cultivation [81]. The loss of the *vlsE* expression locus could explain why K78 cannot stably infect mice. The *vlsE* gene and the partially sequenced *vls* locus with 8 cassettes of PKo (S7 Fig.) are located on the lp28–8 plasmid as in K78 [18]. For ACA-1 and the *B*. *garinii* strain Ip90 the complete sequences for the *vls* locus with 14 and 11 silent *vls*-cassettes, respectively, are both located on plasmid lp28–1 (Genbank accession AY100628 and AY100633, respectively) [116]. The presence of 15 *vls*-cassettes in B31 indicates that the number of silent cassettes in *B*. *afzelii* is most likely lower than in *B*. *burgdorferi* (S7 Fig.).

## Conclusion

The genome sequence of the *B*. *afzelii* strain K78 increases the number of known *B*. *afzelii* chromosomal genomes to five and enables comparative plasmid sequence analysis for three *B*. *afzelii* strains. There is an increasing interest to understand the underlying causes of the different manifestations of Lyme borreliosis and the molecular reasons determining tissue specificity, in relation to gene variation and the presence or absence of certain genes. The availability of multiple complete genome sequences is a prerequisite to perform these kinds of analyses. While a broad basis of genomic data, including a detailed description of the plasmids, has become available for *B*. *burgdorferi*, the only causative agent of LB in the United States, the more heterogeneous landscape of *Borrelia* species associated with Lyme borreliosis in Europe is less well studied. This is true even for the most prevalent species *B*. *afzelii* and *B*. *garinii*, but especially for the availability of plasmid sequence information. The *B*. *afzelii* strain K78, as described here is rather similar to the other *B*. *afzelii* genomes (PKo, ACA-1, Tom3107 and HLJ01) suggesting a high homogeneity within *B*. *afzelii*. A comparison of two *B*. *afzelii* genomes (PKo and ACA-1) with the genomes of 14 *B*. *burgdorferi* and 2 *B*. *garinii* genomes, did not identify any genes which were uniquely present in the *B*. *afzelii* strains [67]. Therefore, *B*. *afzelii* host specificity and tropism have been suggested to be determined by sequence variation, variable numbers of paralogous genes or different expression patterns rather than absence or presence of specific genes. The inclusion of the chromosomes of the Russian strain Tom3107 and the Chinese strain HLJ01 into the comparison allowed for the analysis of conservation with *B*. *afzelii* strains from Asia. However, it can be expected that more significant differences will be seen on the plasmid level, when more of those sequences will become available.

As in *B*. *burgdorferi* [67] and *B*. *garinii* [15], the *B*. *afzelii* chromosome and the circular plasmid families cp32 and cp26 as well as the linear plasmid lp54 (except the PFam54 gene array) are the evolutionary more stable components of the genomes. In contrast, the linear plasmids seem to be evolutionary more unstable and have undergone more re-organization and therefore contain a higher number of degraded genes. Many of the host specific proteins (e.g. PFam54 paralogous family, DbpA, DpbB, Bdr, CRASP-1 etc.) are located on these variable plasmids and many belong to different paralogous gene families with numerous members, which may reflect the reservoir of genes needed for adaptation to a changing environment and a multitude of hosts (98]. The data for K78 give an insight into plasmid variability within *B*. *afzelii* strains and may be of help to further elucidate the molecular mechanisms of the *B*. *afzelii* specific manifestations of Lyme borreliosis.

## Supporting Information

S1 Fig Alignments of the chromosomal contigs. The *B*. *afzelii* strains K78, ACA-1, PKo, Tom3107, HLJ01 and *B*. *burgdorferi* B31 show a high degree of sequence conservation and synteny over the complete length of the chromosomes. Please note that for strain ACA-1 only two unconnected contigs are available, separated at the position of the red line. The contigs were aligned with the “progressiveMauve” aligner of Mauve 2.0 (43) and colored with the “backbone” color scheme showing homologous regions in pink (mauve), or in differing colors when homology is only within a subset of the aligned sequences. The location of the genes are shown with boxes (top/down is direct/indirect strand).
(TIF)

S2 Fig Genome representation of the *B*. *afzelii* K78 chromosome and linear plasmids. From top to bottom are shown: Two plots of GC%, GC skew (colors differentiate values above and below mean), gene positions of RNA and coding sequences, for the direct (above the line) and indirect strand (below the line) and COG functional classification (figures generated with DNAPlotter, Sanger).
(PDF)

S3 Fig Circular genome views of the plasmids. Circular genome plot (CGview) representation of the circular plasmids of *Borrelia afzelii* K78. From the inner circles to the outer circles the nucleotide position, GC skew, GC%, COG classification and gene positions of the indirect strand and gene positions and COG classification of the direct strand are shown. The position of the plasmid partitioning genes *parA* (PFam32) can be traced by searching the brown COG bars.
(PDF)

S4 Fig Inter-organism alignments of the contigs of the circular plasmids and lp54. Alignments of the sequence of *B*. *afzelii* strain K78 with the corresponding plasmids in ACA-1, PKo, Tom3107 (lp54 and cp26) and *B*. *burgdorferi* B31, with the “progressiveMauve” alignment method of Mauve (43). Alignments are colored with the ‘backbone’ coloring scheme of Mauve with homologous parts colored in pink (mauve). The filling of the boxes represents a similarity plot. Default parameters have been used unless stated explicitly otherwise in the figure captions. A: lp54 (“A”), B: cp26 (“B”), C: cp32–9 (“N”), D: cp32–4 (“R”), E: cp32–3 (“S”).
(TIF)

S5 Fig Alignment of the plasmid groups cp32 and lp28 within *Borrelia afzelii* K78. (A) cp32–3, -4, -5 and -9 (“S, R, V, N”) DNA sequences. The similarity plot shows high homology (colored mauve) between the four cp32-type plasmids and a high synteny for the gene composition. Variations are restricted to mainly five small regions. (B) lp28–1, -2, -3 and-4 (“F, G, H, I”) DNA sequences. The split-up of regions, the variations in order and length of the syntenic segments indicates that the linear lp28 plasmids are in a process of dynamic change. Also, many genes on these plasmids are only present in form of fragments/pseudogenes. Yellow boxes indicate the position of the plasmid replication/partitioning genes.
(TIF)

S6 Fig Inter-organism alignments of the contigs of the linear plasmids. Alignments of the K78 linear plasmids (except lp54) with the corresponding plasmid sequences in ACA-1, PKo and *B*. *burgdorferi* B31 generated with the “progressiveMauve” alignment method of Mauve (43). The alignments generally show a higher degree of diversity with gene shuffling or reversed elements than the circular plasmids. The Locally Collinear Blocks (LCBs) coloring scheme has been applied marking related LCBs with the same color. The filling of the boxes shows a similarity plot. Default parameters have been used unless stated explicitly otherwise. (A) lp17, for *B*. *burgdorferi* B31 only the blue LCB of lp17 is oriented in the same direction compared to the LCBs of the shown *B*. *afzelii* strains. It is the 3’-terminal part which shows more diversity in the *B*. *afzelii* strains and is also shortened and less homologous in the *B*. *burgdorferi* B31 strain. (B) lp28–1 (“F”) shows homology to strain ACA-1 over a large portion, but no sequence is available for PKo. B31 lp28–1 which contains the *vls* cassettes does only show partial similarity to the *B*. *afzelii* sequences, whereas generally B31 lp28–2 has a better match. (C) lp28–2 (“G”). The sequences of strains PKo and B31 are shown as their reverse complement to better match the K78, ACA-1 sequences. It is to note that B31 lp28–1 also does not show a good match to the *B*. *afzelii* lp28–2 sequences. To increase sensitivity the “seed-family”-option has been set for the shown”progressiveMauve” alignment. D: lp28–3 (“H”). The sequences of strains PKo and B31 are shown as their reverse complement to better match the K78 and ACA-1 sequences. E: lp28–4 (“I”). The sequence of strain PKo is given as reverse complement. F: lp38 (“J”). It is to note that fragments of *B*. *afzelii* lp38 show similarities to B31 lp28–4.
(TIF)

S7 Fig Dot matrix plots (SeqTools Saenger) for comparison of the *vls* cassettes. For easier comparison the plots have been adapted to show the sequences in the same orientations as for strain K78. (A) *B*. *afzelii vls*. The K78 cassettes *vls4* and *vls6* contain frameshifts (marked with arrows), *vls11* is fragmented. The cassettes in the dot matrix plots are compared to *vls1*. In PKo *vls6* contains a frameshift, *vls3* and *4* are fragmented, and *vls1* appears truncated due to incomplete sequencing. A *vlsE* representative of PKo is taken as reference for the dot matrix plot. For ACA-1 only partial *vlsE* sequences are available, and for the dot matrix plot the ACA-1 silent cassettes are shown compared to *vlsE* of strain PKo. ACA-1 *vls9* contains a frameshift and *vls14* is fragmented. (B) The *B*. *burgdorferi* B31 *vls* locus. Here *vlsE* is adjacent to the silent *vls* cassettes but encoded on the direct strand.
(TIF)

S1 Table Overview of the cellular localization of the proteins . Cellular localization of the predicted proteins on the chromosomes and plasmids in the three *B*. *afzelii* strains K78, ACA-1 and PKo and the *B*. *burgdorferi* strain B31 using the PSORTb 3.0 program (50).
(DOCX)

S2 Table Paralogous genes in *B*. *afzelii* strains K78, PKo, ACA-1 and *B*. *burgdorferi* strain B31. The table lists the results of SCPS protein sequence clustering analysis applying the TribeMCL and spectral clustering algorithms (cut-off E-value 0.001) for the *B*. *afzelii* strains K78, PKo and ACA-1, including plasmid data. *B*. *burgdorferi* B31 is included as reference. Mapping of the results to the PFams was done following the naming scheme as defined by Casjens *et al*. for *B*. *burgdorferi* (9, 46). Sequences which are located on replicons of the same type are arranged in rows in the table within a paralogous gene family assignment. Sequences are named according to the GenBank locus-tags. Dashes are used to mark missing entries, which may fall below cut-off or have been taken out from the proteome annotations, e.g. too short proteins or truncated/degraded proteins, or may be missing in the respective organism.
(XLSX)

S3 Table Sequence references and accession numbers. Strain names and accession codes of: (A) Chromosomes and plasmids of the *Borrelia* strains compared in this work, (B) *ospC* (partial) sequences in the tree and network analysis (Fig. 2), (C) Sequences of the cp26 plasmids containing the intergenic region (Fig. 4).
(XLSX)

S4 Table List of tandem repeats in *Borrelia afzelii* K78 and comparison to the genomes of ACA-1, PKo, HLJ01, Tom3107 and *B*. *burgdorferi* B31. Summary of a “Tandem Repeat Finder” analysis showing the major tandem repeats in the chromosome and the plasmids.
(XLSX)
