# Supplementary figures and images for: Complete Genome Sequence of Borrelia afzelii K78 and Comparative Genome Analysis
Source: PLoS One. 2015 Mar 23;10(3):e0120548. doi: 10.1371/journal.pone.0120548 (PMC4370689; doi:10.1371/journal.pone.0120548)

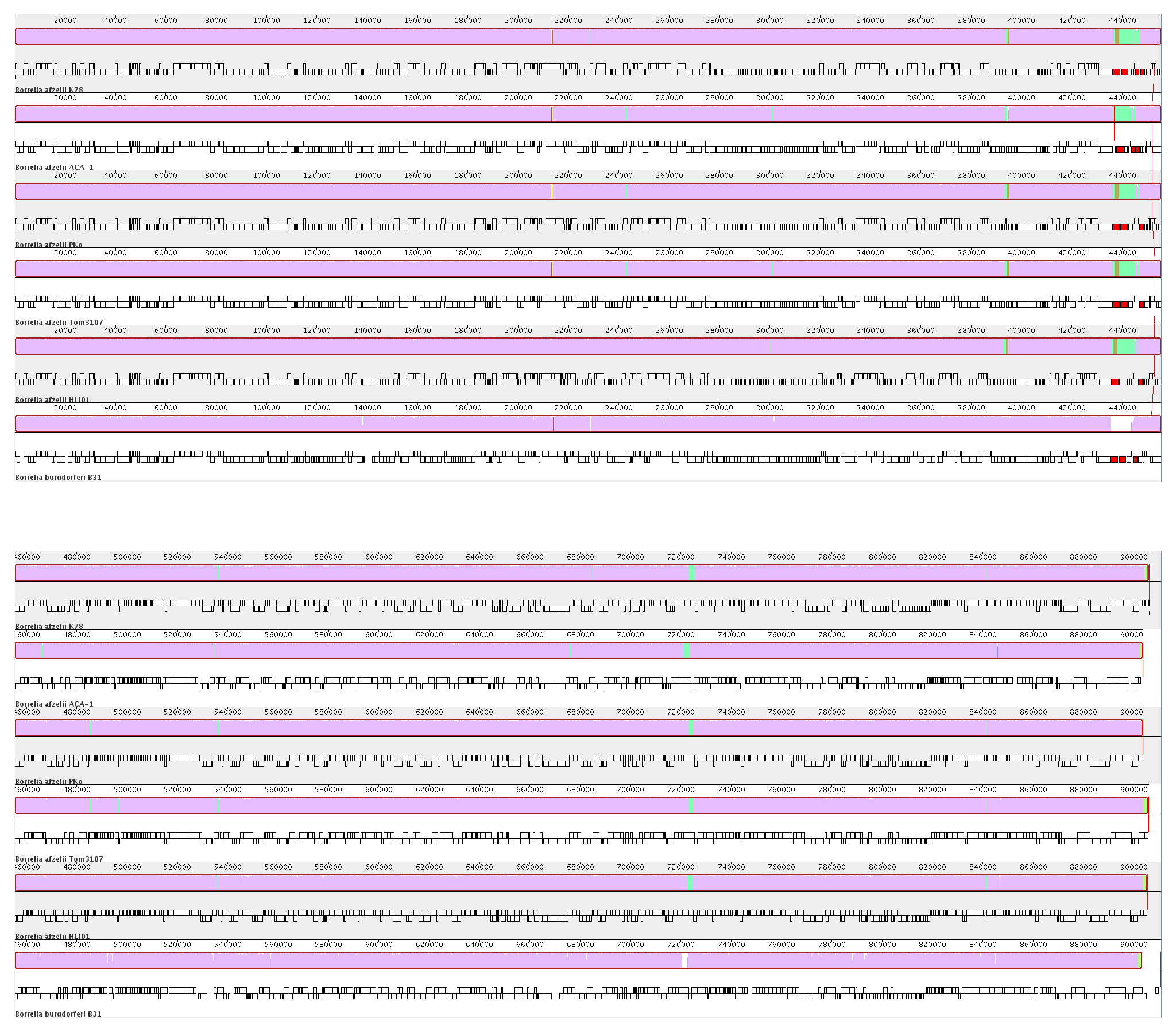

Supplement: S1 Fig — The B. afzelii strains K78, ACA-1, PKo, Tom3107, HLJ01 and B. burgdorferi B31 show a high degree of sequence conservation and synteny over the complete length of the chromosomes. Please note that for strain ACA-1 only two unconnected contigs are available, separated at the position of the red line. The contigs were aligned with the “progressiveMauve” aligner of Mauve 2.0 (43) and colored with the “backbone” color scheme showing homologous regions in pink (mauve), or in differing colors when homology is only within a subset of the aligned sequences. The location of the genes are shown with boxes (top/down is direct/indirect strand). (TIF) [file pone.0120548.s001.tif]

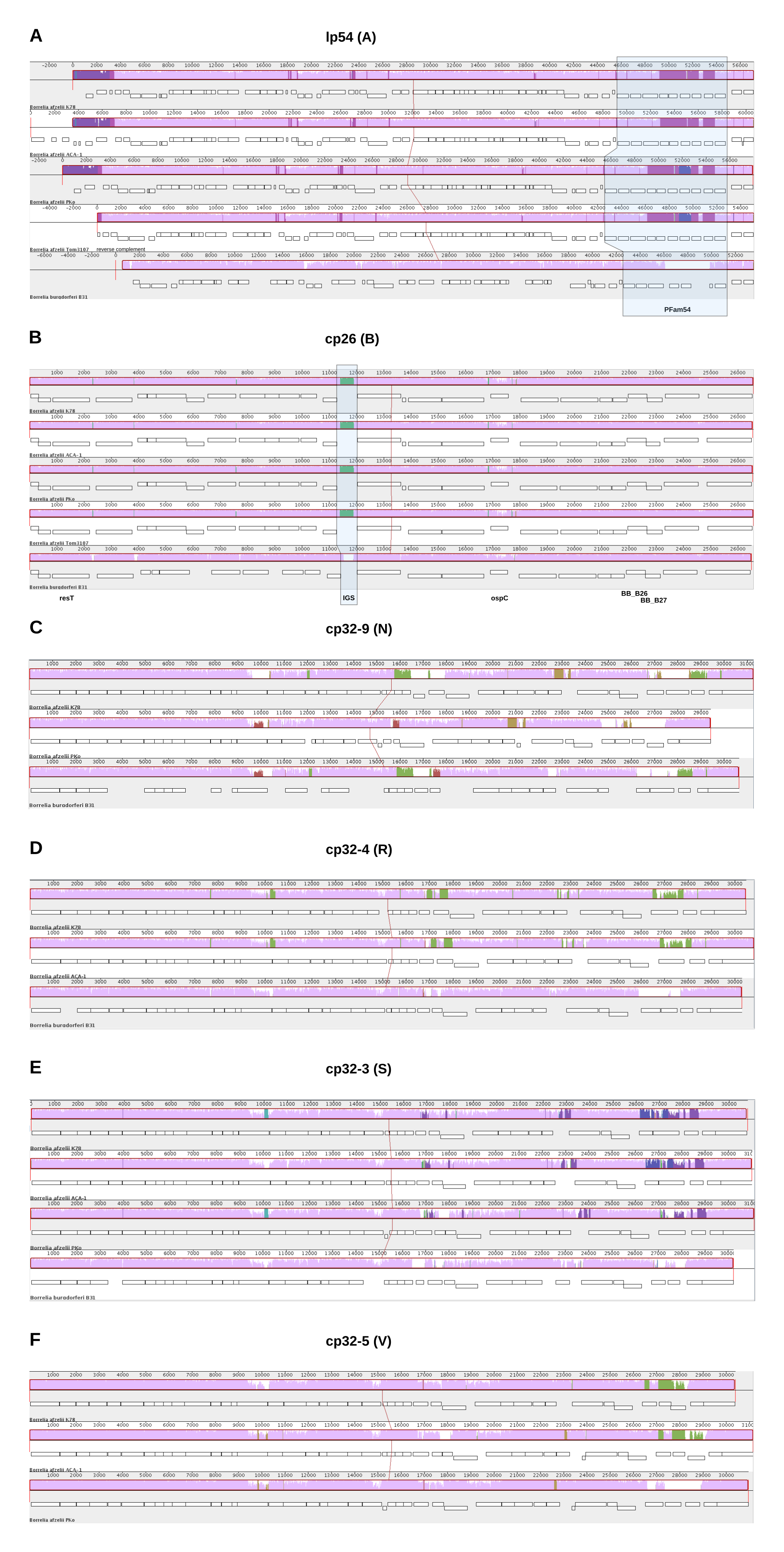

Supplement: S4 Fig — Alignments of the sequence of B. afzelii strain K78 with the corresponding plasmids in ACA-1, PKo, Tom3107 (lp54 and cp26) and B. burgdorferi B31, with the “progressiveMauve” alignment method of Mauve (43). Alignments are colored with the ‘backbone’ coloring scheme of Mauve with homologous parts colored in pink (mauve). The filling of the boxes represents a similarity plot. Default parameters have been used unless stated explicitly otherwise in the figure captions. A: lp54 (“A”), B: cp26 (“B”), C: cp32–9 (“N”), D: cp32–4 (“R”), E: cp32–3 (“S”). (TIF) [file pone.0120548.s004.tif]

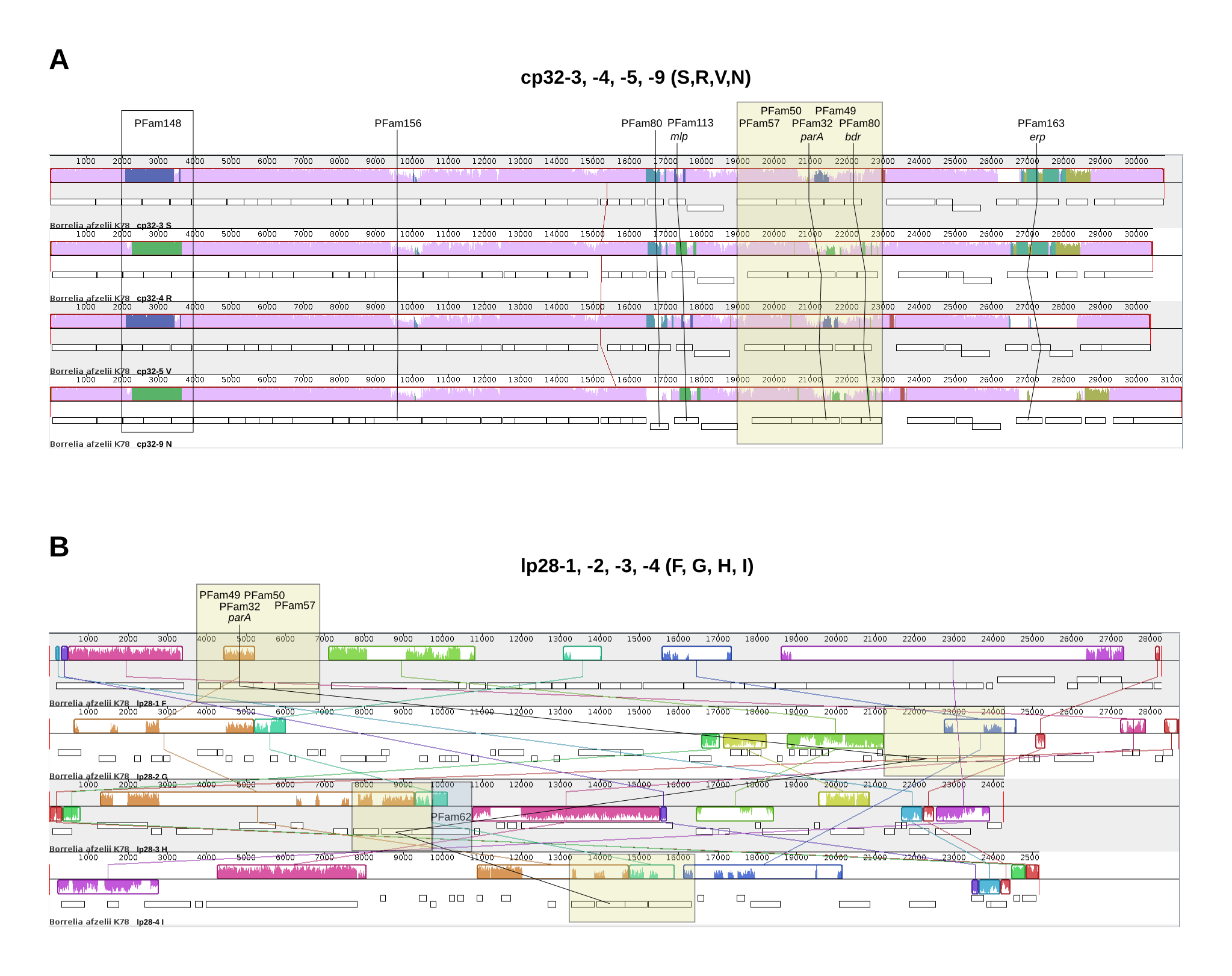

Supplement: S5 Fig — (A) cp32–3, -4, -5 and -9 (“S, R, V, N”) DNA sequences. The similarity plot shows high homology (colored mauve) between the four cp32-type plasmids and a high synteny for the gene composition. Variations are restricted to mainly five small regions. (B) lp28–1, -2, -3 and-4 (“F, G, H, I”) DNA sequences. The split-up of regions, the variations in order and length of the syntenic segments indicates that the linear lp28 plasmids are in a process of dynamic change. Also, many genes on these plasmids are only present in form of fragments/pseudogenes. Yellow boxes indicate the position of the plasmid replication/partitioning genes. (TIF) [file pone.0120548.s005.tif]

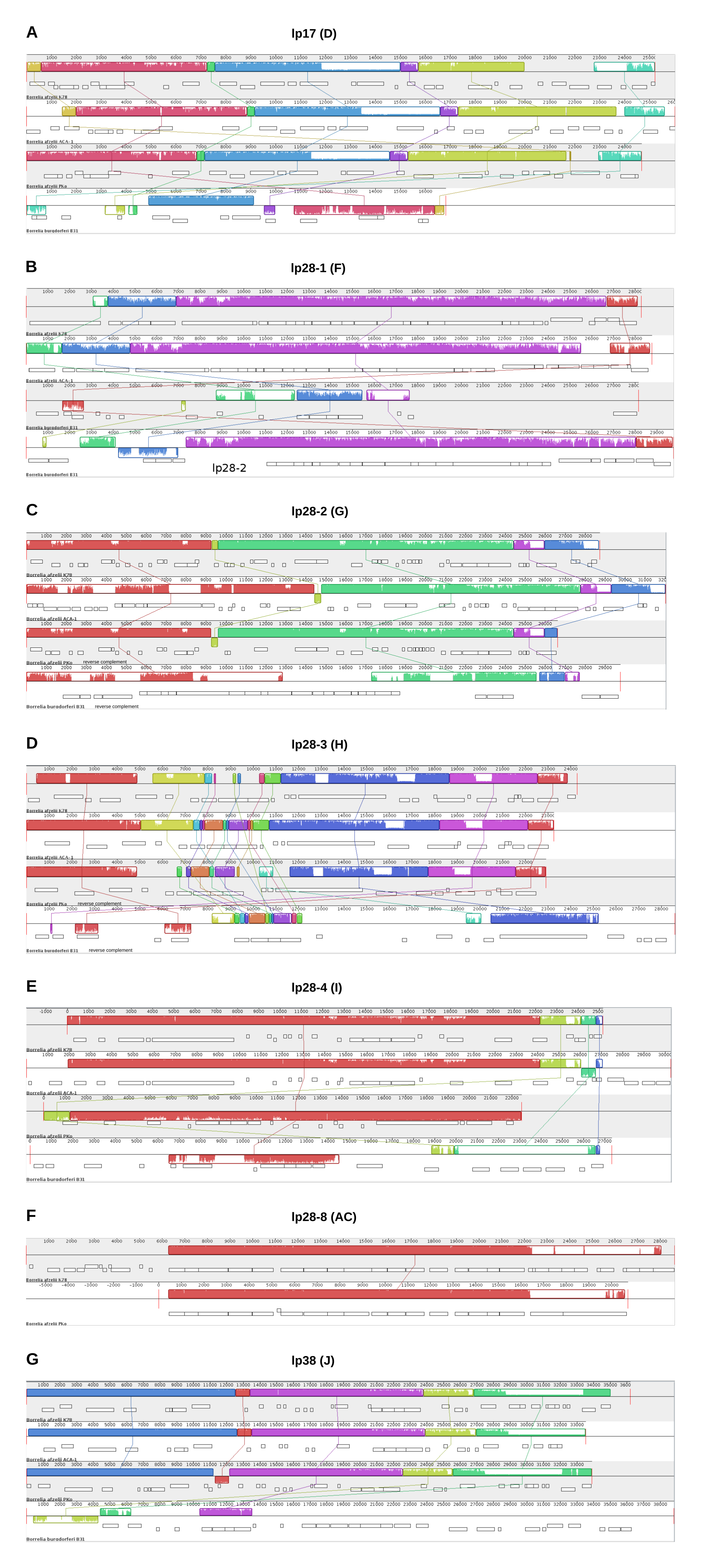

Supplement: S6 Fig — Alignments of the K78 linear plasmids (except lp54) with the corresponding plasmid sequences in ACA-1, PKo and B. burgdorferi B31 generated with the “progressiveMauve” alignment method of Mauve (43). The alignments generally show a higher degree of diversity with gene shuffling or reversed elements than the circular plasmids. The Locally Collinear Blocks (LCBs) coloring scheme has been applied marking related LCBs with the same color. The filling of the boxes shows a similarity plot. Default parameters have been used unless stated explicitly otherwise. (A) lp17, for B. burgdorferi B31 only the blue LCB of lp17 is oriented in the same direction compared to the LCBs of the shown B. afzelii strains. It is the 3’-terminal part which shows more diversity in the B. afzelii strains and is also shortened and less homologous in the B. burgdorferi B31 strain. (B) lp28–1 (“F”) shows homology to strain ACA-1 over a large portion, but no sequence is available for PKo. B31 lp28–1 which contains the vls cassettes does only show partial similarity to the B. afzelii sequences, whereas generally B31 lp28–2 has a better match. (C) lp28–2 (“G”). The sequences of strains PKo and B31 are shown as their reverse complement to better match the K78, ACA-1 sequences. It is to note that B31 lp28–1 also does not show a good match to the B. afzelii lp28–2 sequences. To increase sensitivity the “seed-family”-option has been set for the shown”progressiveMauve” alignment. D: lp28–3 (“H”). The sequences of strains PKo and B31 are shown as their reverse complement to better match the K78 and ACA-1 sequences. E: lp28–4 (“I”). The sequence of strain PKo is given as reverse complement. F: lp38 (“J”). It is to note that fragments of B. afzelii lp38 show similarities to B31 lp28–4. (TIF) [file pone.0120548.s006.tif]

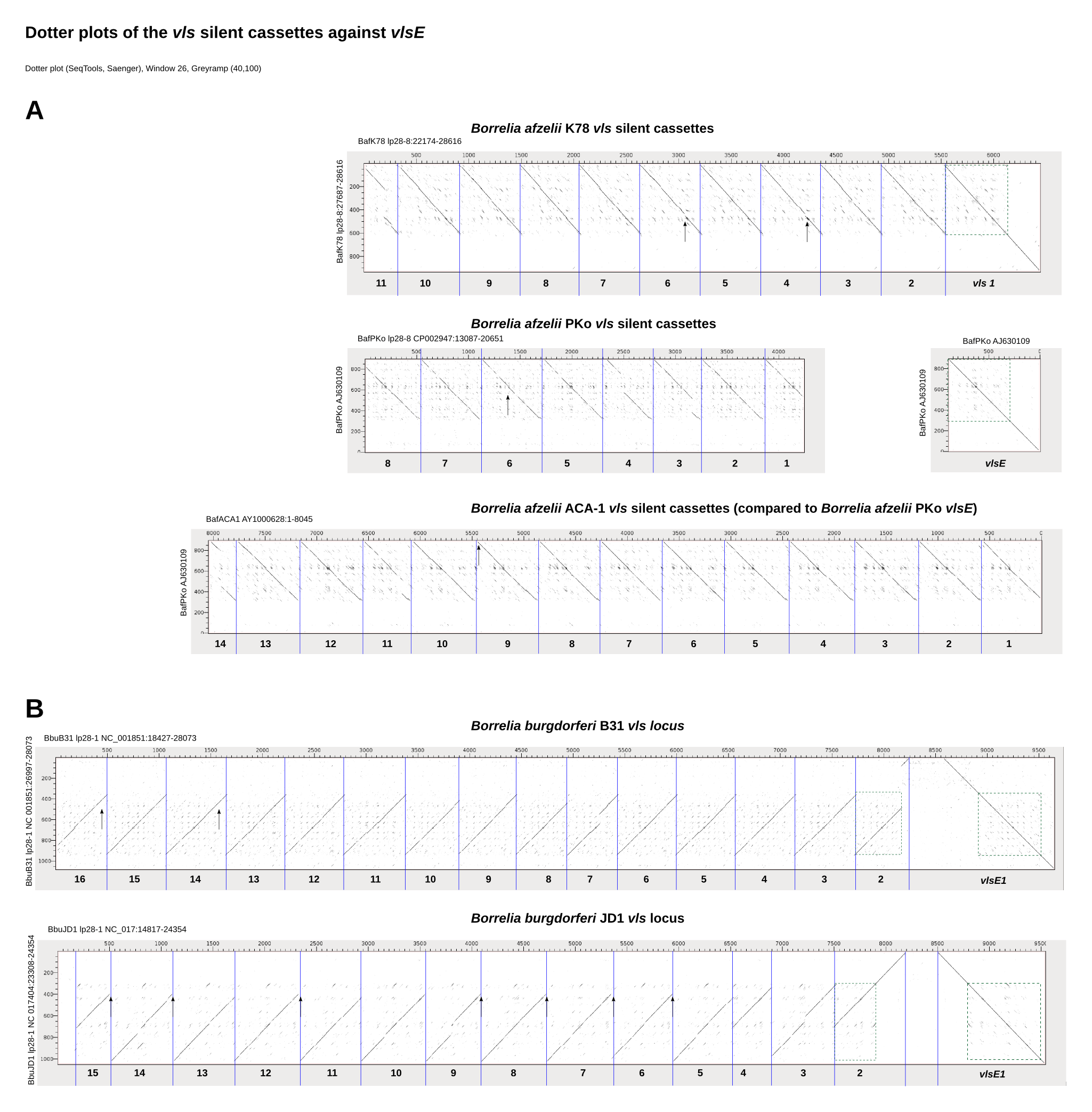

Supplement: S7 Fig — For easier comparison the plots have been adapted to show the sequences in the same orientations as for strain K78. (A) B. afzelii vls. The K78 cassettes vls4 and vls6 contain frameshifts (marked with arrows), vls11 is fragmented. The cassettes in the dot matrix plots are compared to vls1. In PKo vls6 contains a frameshift, vls3 and 4 are fragmented, and vls1 appears truncated due to incomplete sequencing. A vlsE representative of PKo is taken as reference for the dot matrix plot. For ACA-1 only partial vlsE sequences are available, and for the dot matrix plot the ACA-1 silent cassettes are shown compared to vlsE of strain PKo. ACA-1 vls9 contains a frameshift and vls14 is fragmented. (B) The B. burgdorferi B31 vls locus. Here vlsE is adjacent to the silent vls cassettes but encoded on the direct strand. (TIF) [file pone.0120548.s007.tif]
