# Supplementary material for: Complete Genome Sequence of Borrelia afzelii K78 and Comparative Genome Analysis
Source: PLoS One. 2015 Mar 23;10(3):e0120548. doi: 10.1371/journal.pone.0120548 (PMC4370689; doi:10.1371/journal.pone.0120548)

Legends

Tracks (top to bottom):
GC%, GC-skew, RNA fwd/RNA rev, CDS fwd/CDS rev, COG assignment, position (bp)

Plot parameters
GC%, GC-skew: Window size=1000 (plasmids: 400), base step size=20

Color	Graph	Description
	GC%	above mean
	GC%	below mean
	GC skew	above mean
	GC skew	below mean
	RNA	tRNA (top: forward, bottom: reverse)
	RNA	rRNA (top: forward, bottom: reverse)
	RNA	ncRNA, misc_RNA (top: forward, bottom: reverse)
	CDS	forward
	CDS	reverse

Color	COG	Description
	A	RNA processing and modification
	B	Chromatin structure and dynamics
	C	Energy production and conversion
	D	Cell division and chromosome partitioning
	E	Amino acid transport and metabolism
	F	Nucleotide transport and metabolism
	G	Carbohydrate transport and metabolism
	H	Coenzyme metabolism
	I	Lipid metabolism
	J	Translation, ribosomal structure and biogenesis
	K	Transcription
	L	DNA replication, recombination, and repair
	M	Cell envelope biogenesis, outer membrane
	N	Cell motility and secretion
	O	Posttranslational modification, protein turnover, chaperones
	P	Inorganic ion transport and metabolism
	Q	Secondary metabolites biosynthesis, transport, and catabolism
	R	General function prediction only
	S	Function unknown
	T	Signal transduction mechanisms
	U	Intracellular trafficking and secretion
	V	Defense mechanisms
	W	Extracellular structures
	Y	Nuclear structure
	Z	Cytoskeleton

A - Chromosome [*Borrelia afzelii* K78] 905949 bp

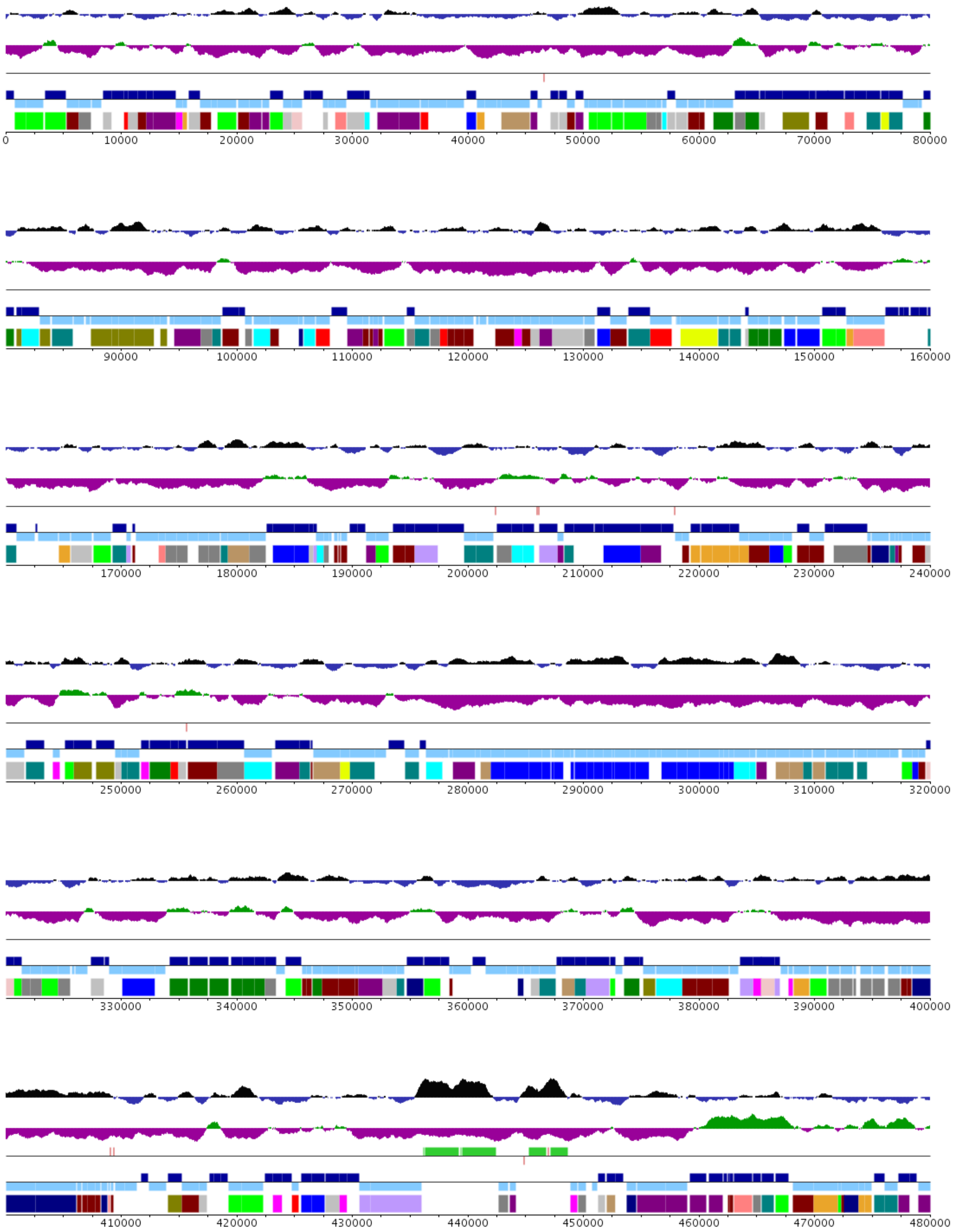

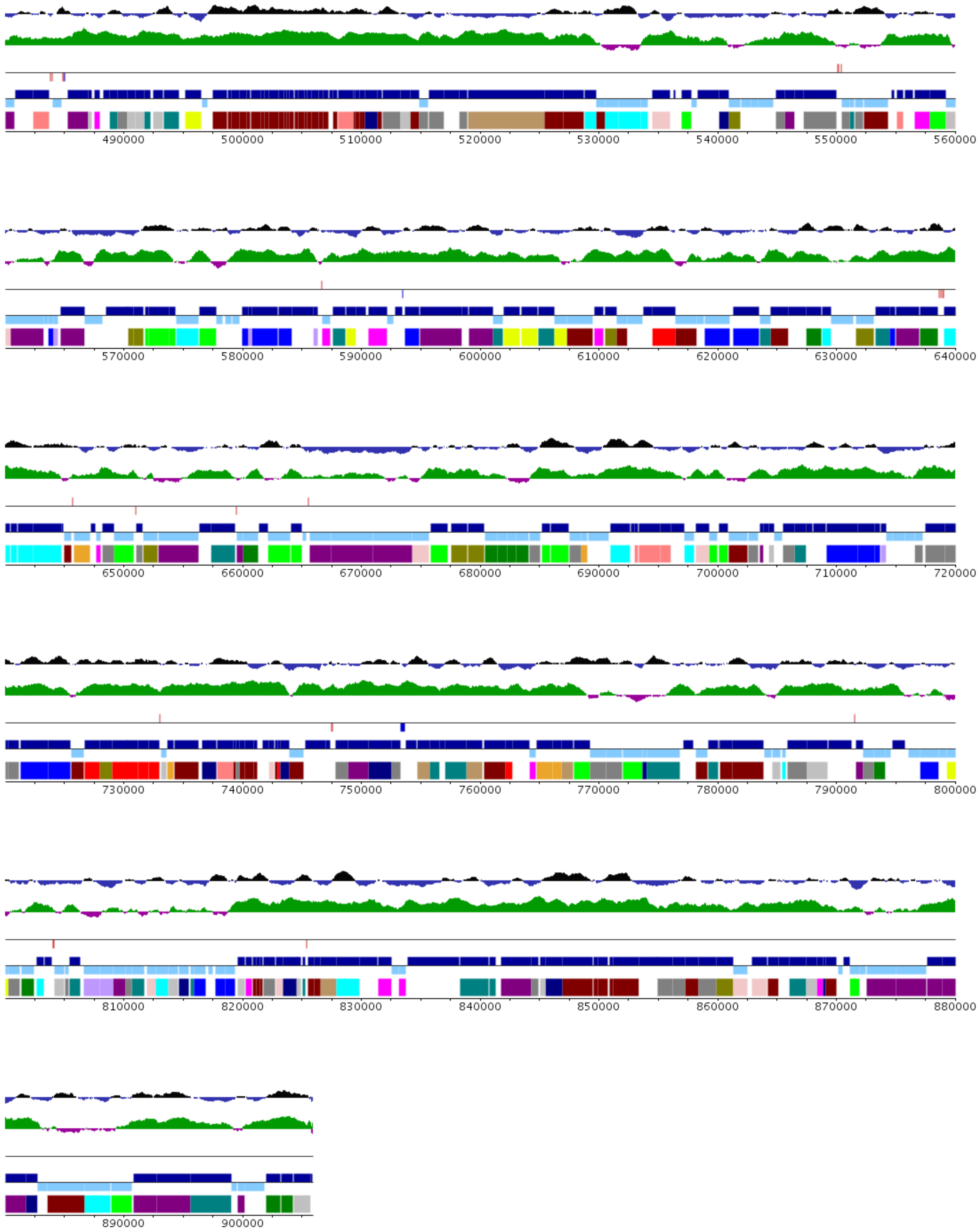
490000
500000
510000
520000
530000
540000
550000
560000
570000
580000
590000
600000
610000
620000
630000
640000
650000
660000
670000
680000
690000
700000
710000
720000
730000
740000
750000
760000
770000
780000
790000
800000
810000
820000
830000
840000
850000
860000
870000
880000
890000
900000

B - Plasmid lp17 “D” 25221 bp

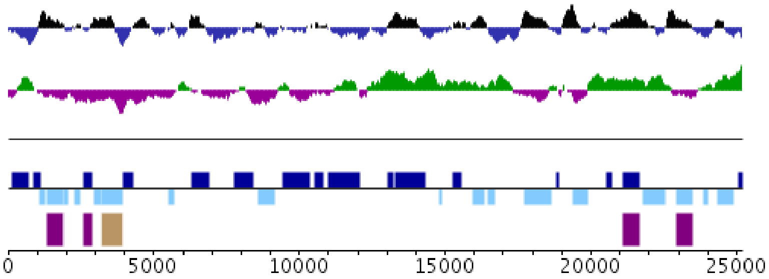

C - Plasmid lp28-1 “F” 28272 bp

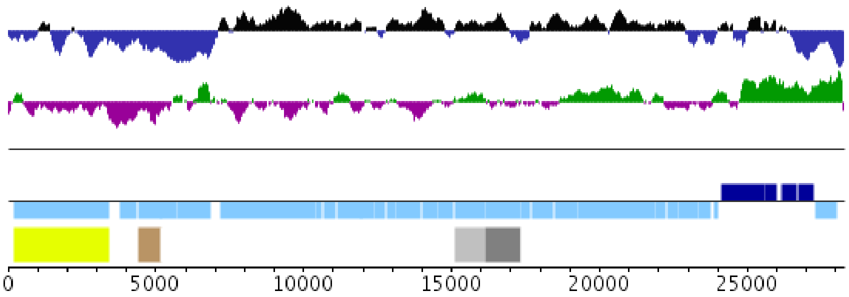

D - Plasmid lp28-2 “G” 28738 bp

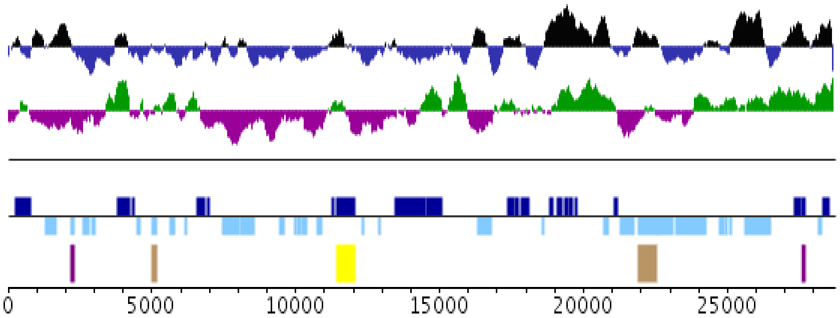

E - Plasmid lp28-3 “H” 24282 bp

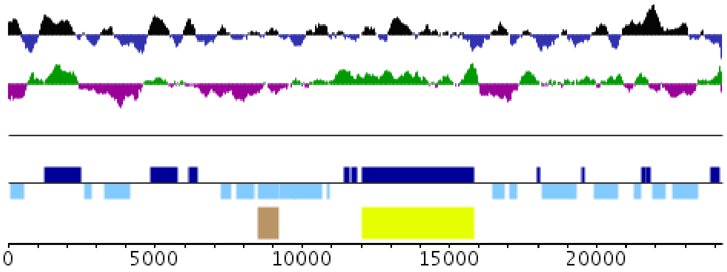

F - Plasmid lp28-4 “I” 25184 bp

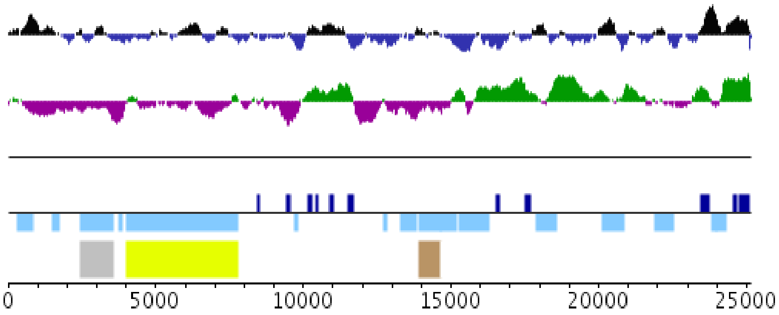

G - Plasmid lp28-8 “AC” 28638 bp

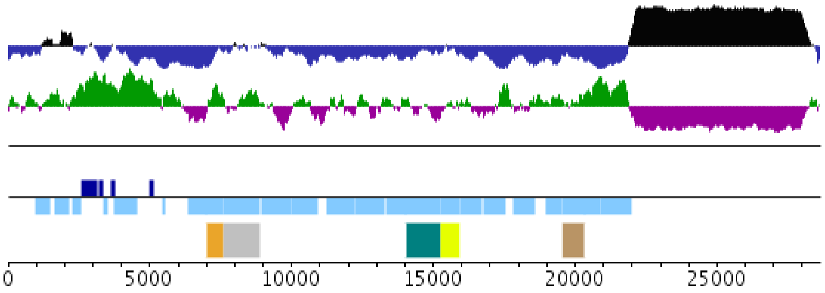

H - Plasmid lp38 “J” 36114 bp

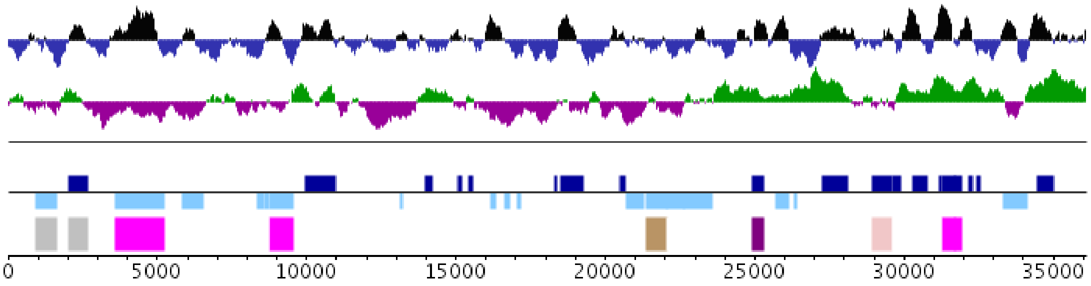

I - Plasmid lp54 “A” 57181 bp

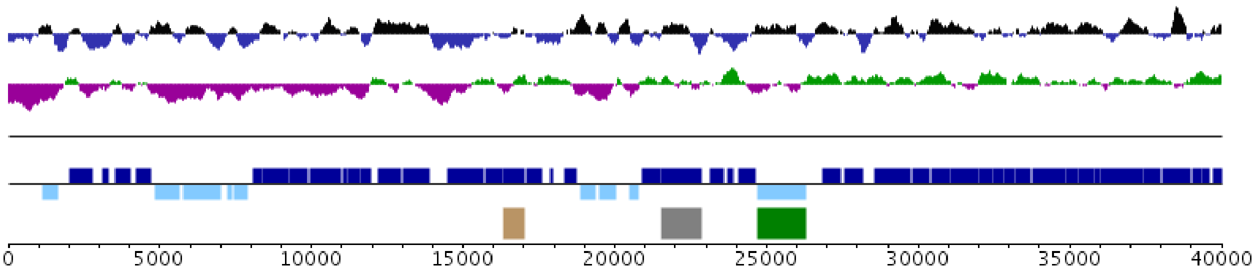

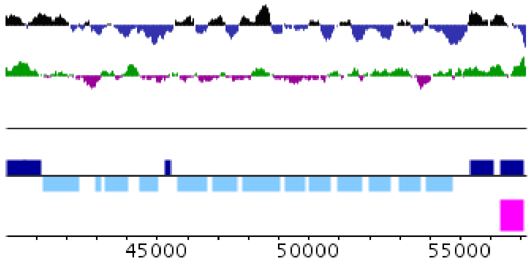

Supplement: S2 Fig — From top to bottom are shown: Two plots of GC%, GC skew (colors differentiate values above and below mean), gene positions of RNA and coding sequences, for the direct (above the line) and indirect strand (below the line) and COG functional classification (figures generated with DNAPlotter, Sanger). (PDF) [file pone.0120548.s002.pdf]
