# Supplementary material for: Complete Genome Sequence of Borrelia afzelii K78 and Comparative Genome Analysis
Source: PLoS One. 2015 Mar 23;10(3):e0120548. doi: 10.1371/journal.pone.0120548 (PMC4370689; doi:10.1371/journal.pone.0120548)

Legends

Tracks (inner to outer):
Position (bp), GC-skew lower/higher mean, GC% lower/higher mean
COG assignment rev, CDS rev, CDS fwd, COG assignment fwd

Color	Graph	Description
	GC%	above mean
	GC%	below mean
	GC skew	above mean
	GC skew	below mean
	RNA	tRNA (top: forward, bottom: reverse)
	RNA	rRNA (top: forward, bottom: reverse)
	RNA	ncRNA, misc_RNA (top: forward, bottom: reverse)
	CDS	forward
	CDS	reverse

Color	COG	Description
	A	RNA processing and modification
	B	Chromatin structure and dynamics
	C	Energy production and conversion
	D	Cell division and chromosome partitioning
	E	Amino acid transport and metabolism
	F	Nucleotide transport and metabolism
	G	Carbohydrate transport and metabolism
	H	Coenzyme metabolism
	I	Lipid metabolism
	J	Translation, ribosomal structure and biogenesis
	K	Transcription
	L	DNA replication, recombination, and repair
	M	Cell envelope biogenesis, outer membrane
	N	Cell motility and secretion
	O	Posttranslational modification, protein turnover, chaperones
	P	Inorganic ion transport and metabolism
	Q	Secondary metabolites biosynthesis, transport, and catabolism
	R	General function prediction only
	S	Function unknown
	T	Signal transduction mechanisms
	U	Intracellular trafficking and secretion
	V	Defense mechanisms
	W	Extracellular structures
	Y	Nuclear structure
	Z	Cytoskeleton

A - *Borrelia afzelii* K78 plasmid cp26 “B”

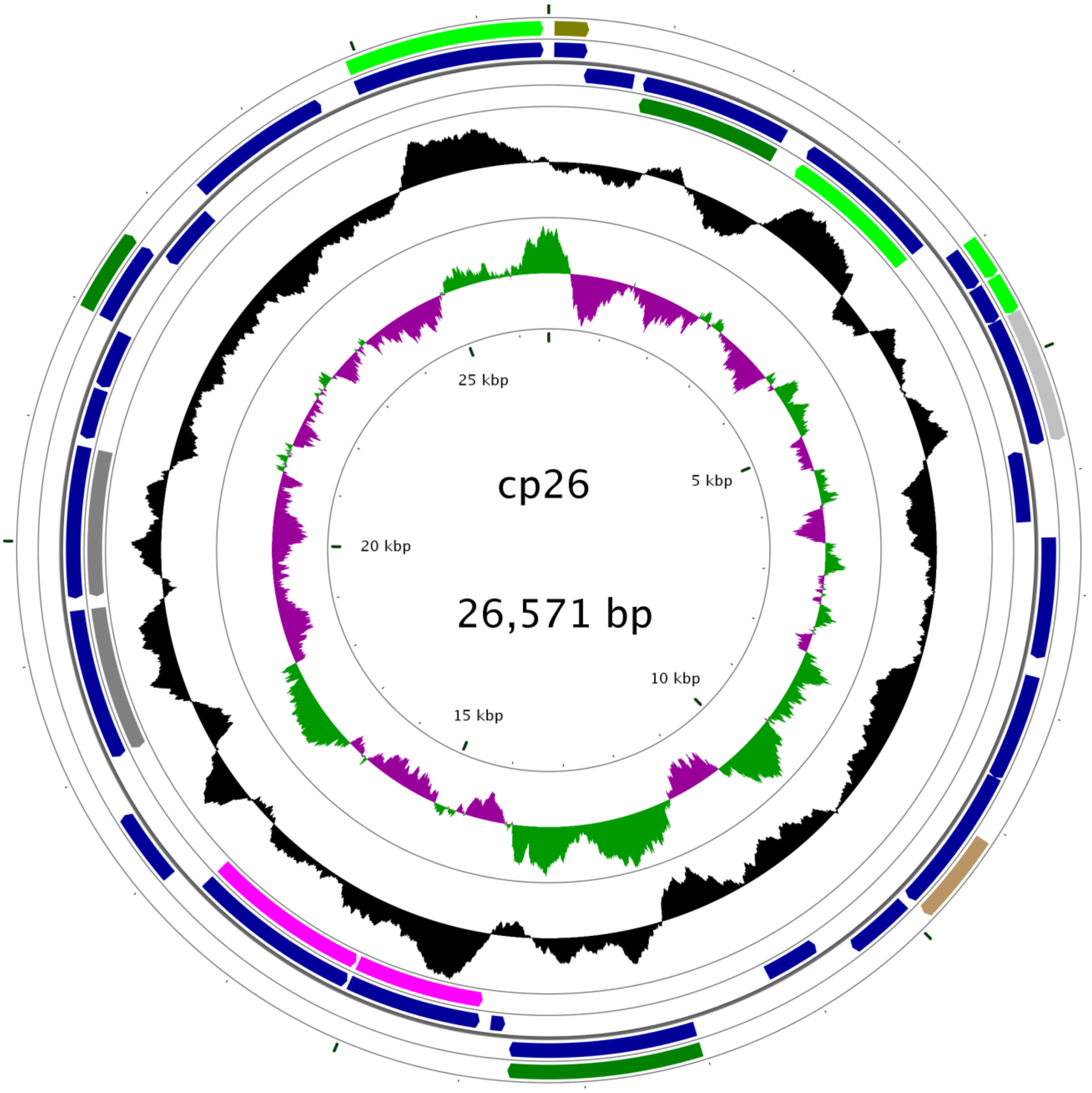

B - *Borrelia afzelii* K78 plasmid cp32-3 “S”

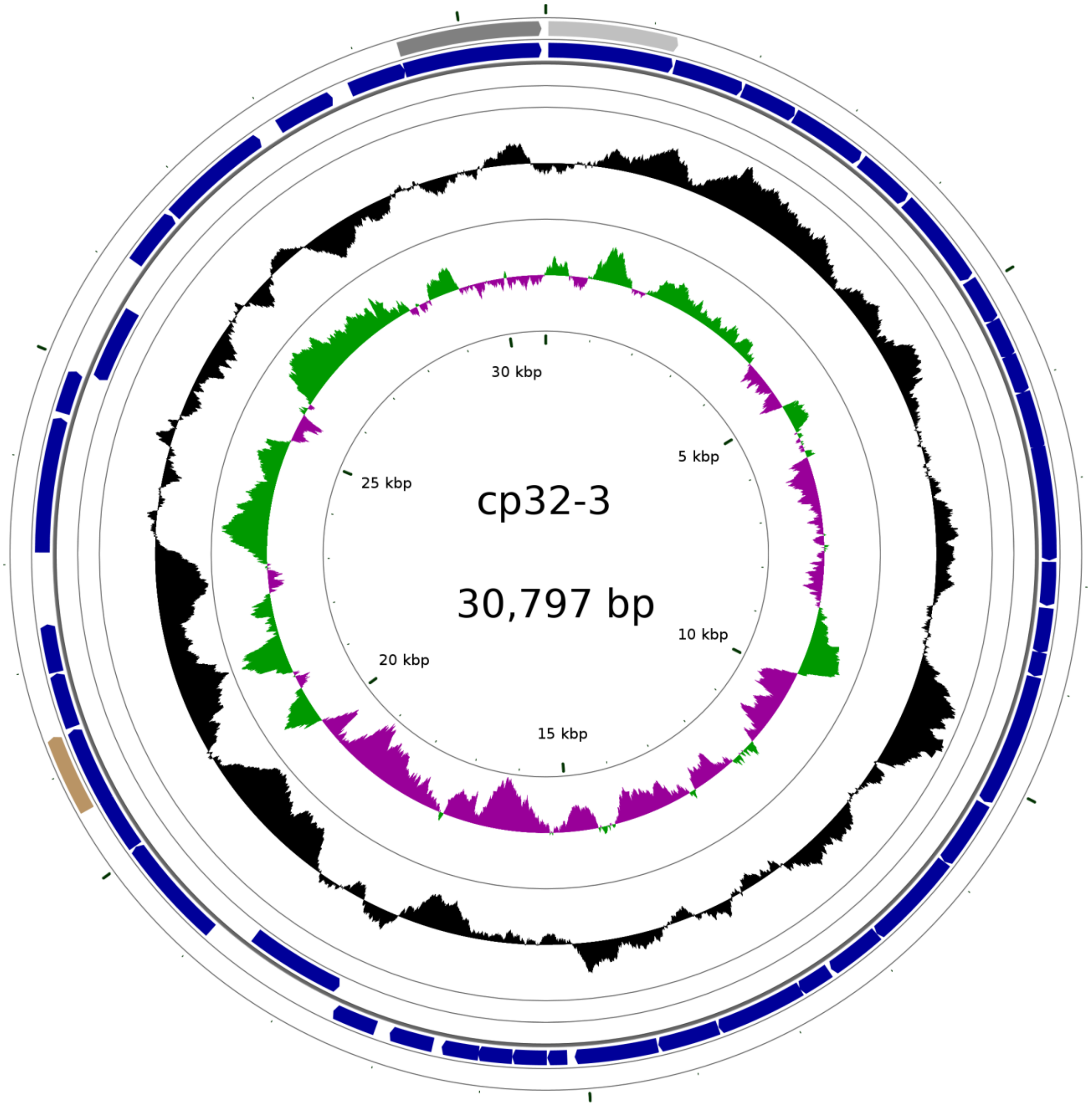

C - *Borrelia afzelii* K78 plasmid cp32-4 "R"

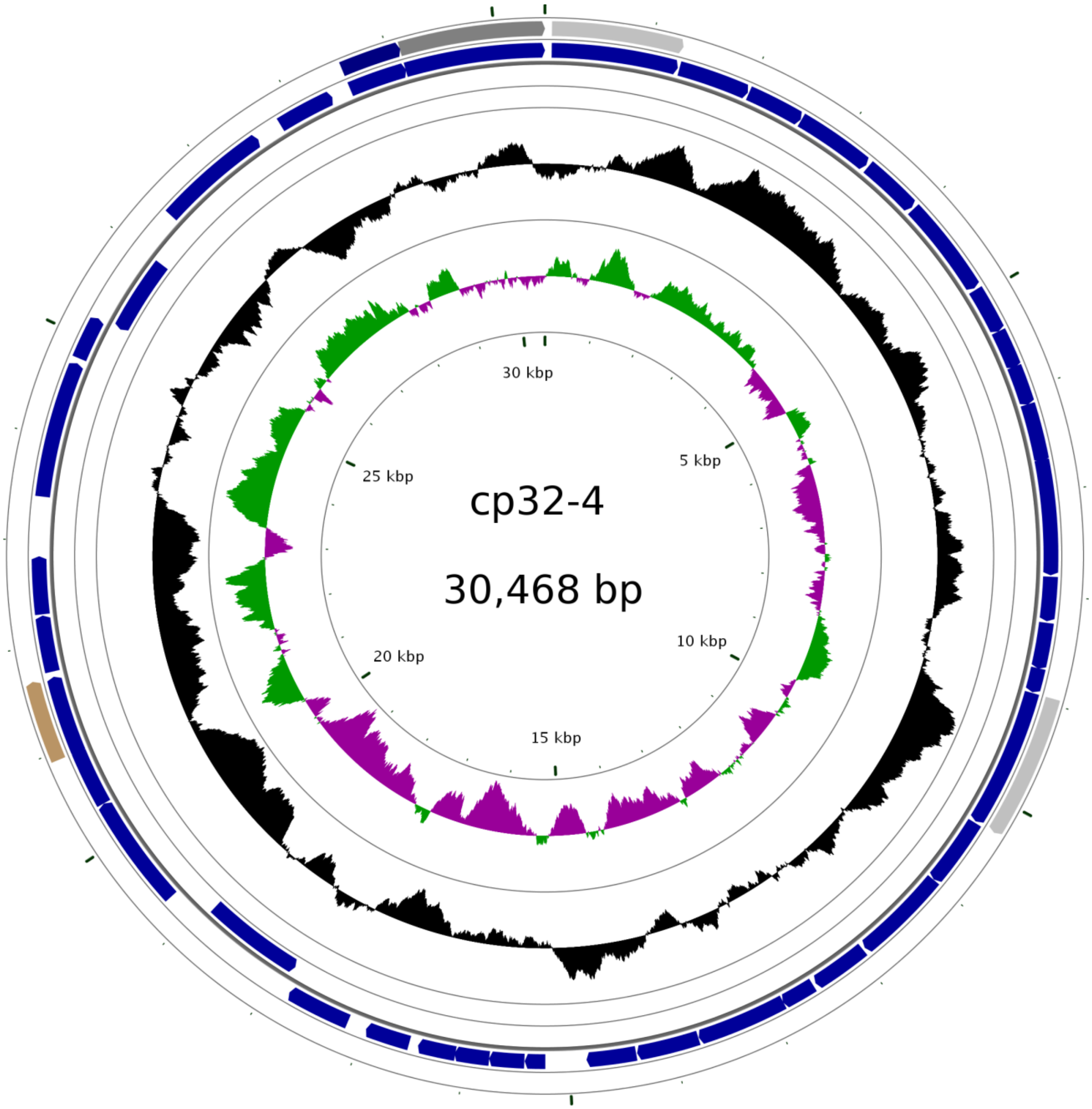

D - *Borrelia afzelii* K78 plasmid cp32-5 "V"

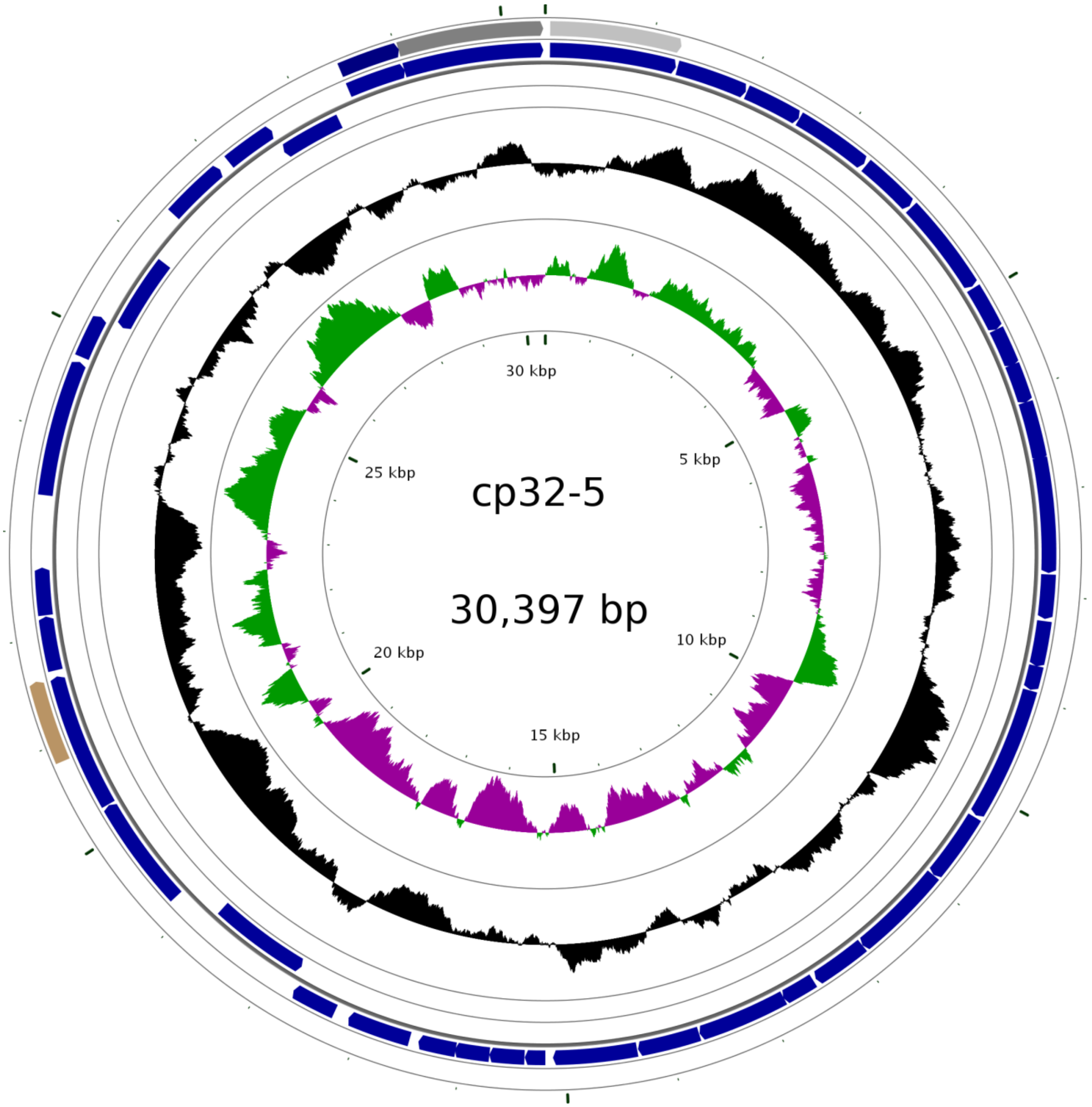

E - *Borrelia afzelii* K78 plasmid cp32-9 "N"

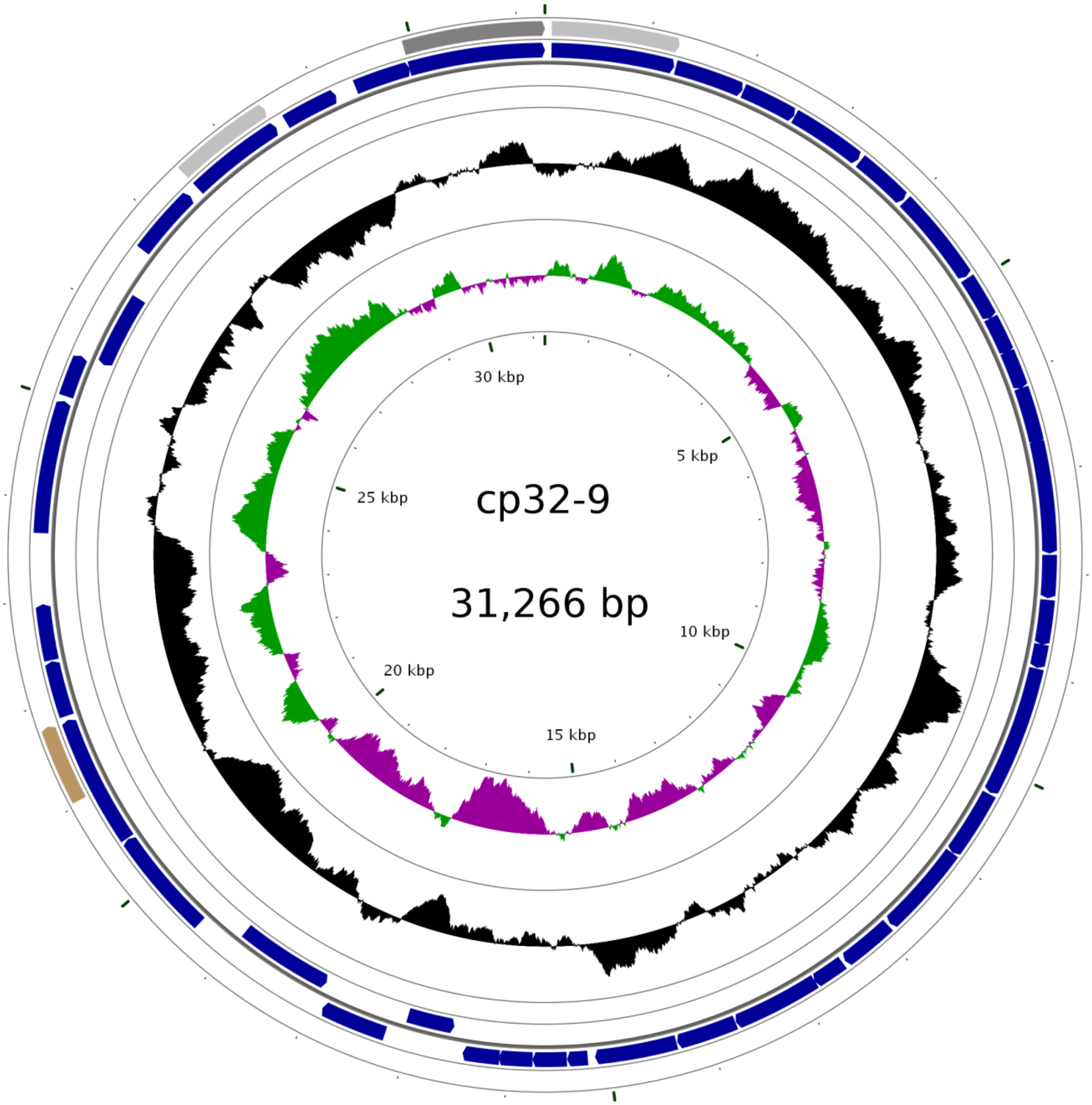

Supplement: S3 Fig — Circular genome plot (CGview) representation of the circular plasmids of Borrelia afzelii K78. From the inner circles to the outer circles the nucleotide position, GC skew, GC%, COG classification and gene positions of the indirect strand and gene positions and COG classification of the direct strand are shown. The position of the plasmid partitioning genes parA (PFam32) can be traced by searching the brown COG bars. (PDF) [file pone.0120548.s003.pdf]
