# Supplementary material for: Complete Genome Sequence of Borrelia afzelii K78 and Comparative Genome Analysis
Source: PLoS One. 2015 Mar 23;10(3):e0120548. doi: 10.1371/journal.pone.0120548 (PMC4370689; doi:10.1371/journal.pone.0120548)
Supplement: S1 Table — . Cellular localization of the predicted proteins on the chromosomes and plasmids in the three B. afzelii strains K78, ACA-1 and PKo and the B. burgdorferi strain B31 using the PSORTb 3.0 program (50). (DOCX) [file pone.0120548.s008.docx]

Overview of the cellular localization of the proteins annotated on the chromosomes and plasmids in the three *B. afzelii* strains K78, ACA-1 and PKo and the *B. burgdorferi* strain B31.

| **Chromosome** | **Baf K78** | **Baf ACA-1** | **Baf PKo** | **Bbu B31** |
| --- | --- | --- | --- | --- |
| Cytoplasmic | 462 | 450 | 458 | 454 |
| Cytoplasmic Membrane | 161 | 155 | 160 | 156 |
| Extracellular | 6 | 6 | 6 | 5 |
| Outer Membrane | 22 | 21 | 18 | 19 |
| Periplasmic | 11 | 11 | 11 | 8 |
| Unknown | 152 | 155 | 171 | 155 |

| **Plasmids** | **Baf K78** | **Baf ACA-1** | **Baf PKo** | **Bbu B31** |
| --- | --- | --- | --- | --- |
| Cytoplasmic | 183 | 223 | 230 | 250 |
| Cytoplasmic Membrane | 30 | 23 | 23 | 32 |
| Extracellular | 0 | 0 | 0 | 1 |
| Outer Membrane | 9 | 5 | 8 | 5 |
| Periplasmic | 1 | 1 | 1 | 2 |
| Unknown | 273 | 298 | 368 | 303 |

Cellular localization was predicted using the PSORTb 3.0 program.
